# Effect of the Entanglement of Microporous Pillared MOFs on the Uptake and Release Profiles of Essential Oil Components

**DOI:** 10.1002/chem.202501167

**Published:** 2025-06-04

**Authors:** Dario Giovanardi, Erika Ribezzi, Marta Napolitano, Martina Orlandini, Nicolò Riboni, Paolo Pio Mazzeo, Alessia Bacchi, Federica Bianchi, Maria Careri, Paolo Pelagatti

**Affiliations:** ^1^ Department of Chemical Sciences, Live Science and Environmental Sustainability University of Parma Parco Area delle Scienze 17/A Parma 43124 Italy; ^2^ Interuniversity Consortium of Chemical Reactivity and Catalysis (CIRCC) Via Celso Ulpiani 27 Bari 70126 Italy; ^3^ Interdepartmental Center for Packaging Tecnopolo, Padiglione 33, Campus Universitario Parma 43124 Italy; ^4^ Biopharmanet‐tec Parco Area delle Scienze 27/A Parma 43124 Italy

**Keywords:** controlled guest release, guest inclusion, host‐guest systems, HS‐GC‐MS, pillared MOFs

## Abstract

Metal‐organic frameworks (MOFs) are a class of coordination polymers known for their intrinsic porosity. This property, combined with their ability to exhibit some degree of flexibility, makes them ideal materials for various innovative applications, like the inclusion and controlled release of active compounds for active packaging and agricultural applications. This study aimed to characterize the uptake and release behavior of an equimolar mixture of two phenolic essential oils (EOs), that is, carvacrol (CAR) and thymol (THY), encapsulated in two microporous Zn‐based MOFs with different entanglement motifs: PUM168 is threefold interpenetrated and flexible, while PUM210 is fourfold polycatenated and rigid (PUM = Parma University Materials). The guest uptake was calculated using Nuclear Magnetic Resonance (^1^H NMR) spectroscopy and thermal gravimetric analysis (TGA) analysis, whereas the release profiles were investigated via headspace gas chromatography‐mass spectrometry (HS‐GC‐MS) (Headspace‐Gas Chromatography coupled with Mass‐Spectrometry) analysis over a period of 15 days at room temperature. The monitoring revealed the superior ability of the flexible PUM168 for a stable and prolonged release of the volatile components, while the release from the rigid PUM210 was completed within 24 hours.

## Graphical abstract

Two differently entangled pillared MOFs with similar chemical compositions but different flexibility were compared for their guest uptake/release capacity. The flexible interpenetrated MOF shows superior host‐guest propensity with respect to the polycatenated one.

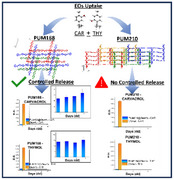

## 1 Introduction

**Figure 1:**
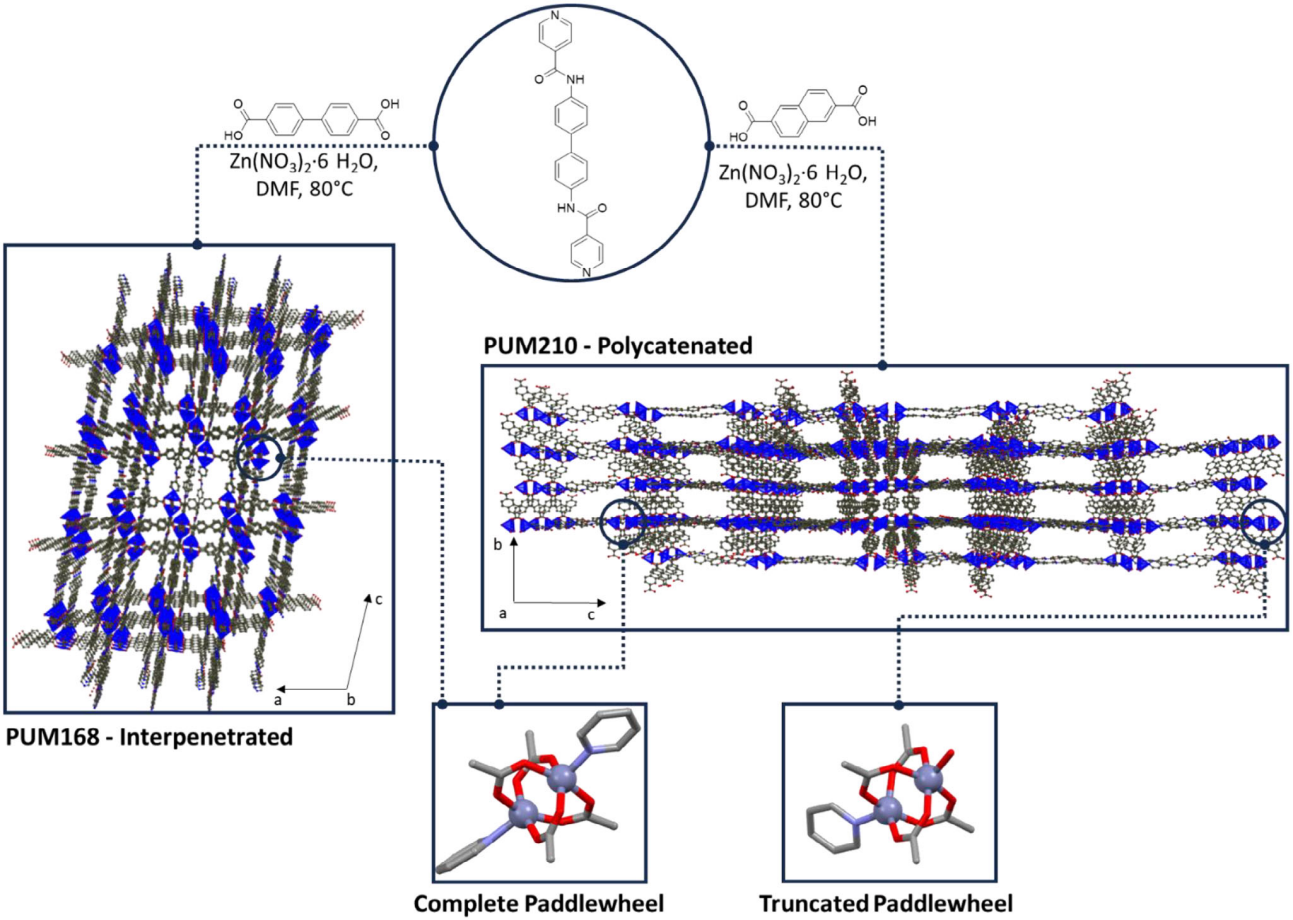
Synthesis of PUM168 and PUM210 with the perspective views of the corresponding frameworks: threefold interpenetration for PUM168 (left) and fourfold polycatenation for PUM210 (right). The paddle‐wheel secondary‐building‐units (SBUs) are represented as blue polyhedral, while the organic linkers are reported in ball‐and‐stick style. The square insets contain the representations of the SBUs found in the two MOFs; color code: O red, N blue, Zn purple, C gray. A comparative figure highlighting the entanglements and potential void volumes of the frameworks is reported in the Supporting Information (Figure S11).

The inclusion of active components in metal‐organic frameworks (MOFs) and their subsequent controlled release is one of the most investigated topics due to its potential for practical real‐world applications.^[^ ^1^ ^]^ The ordered distribution of voids in crystalline MOFs enables the organized inclusion of guest molecules within the framework, and it is expected to positively impact the guest release profile. Notably, when the crystal framework allows guest accommodation without damaging the crystal, via a single‐crystal to single‐crystal transformation (SCSC), the loaded crystal can be interrogated by an X‐ray beam for structural elucidation. This approach, inspired by the crystalline sponge method,^[^ ^2^, ^3^ ^]^ is recurrent for flexible MOFs^[^ ^4^ ^]^ and provides a detailed description of the final host‐guest system,^[^ ^5^ ^]^ leading to the structural elucidation of the included guest, the guest‐induced framework rearrangements, and the supramolecular interactions responsible for the guest loading. These interactions play a pivotal role in controlling the subsequent release of the guest. A high degree of order in guest arrangement is achieved when robust host‐guest interactions occur inside the MOF framework. These interactions are dictated by the affinity between the functionalities featuring the guest molecule and the MOF walls. The size of the guest molecule is also crucial, since molecules bigger than the voids present in the MOF will not be easily accommodated unless the framework exhibits sufficient flexibility and adaptive behavior.^[^ ^6^ ^]^ The use of multifunctional MOFs with diverse receptor sites offers the possibility of obtaining selective inclusion and controlled release of active guests. In this regard, mixed‐ligand MOFs are particularly advantageous because the embedding of different linkers into the crystalline framework increases the degree of functionalization.^[^ ^7^, ^8^ ^]^ In our previous studies we have demonstrated that the pillared MOF PUM168 (PUM = Parma University Materials) has superior uptake ability toward naturally occurring phenol derivatives, such as eugenol (EUG), carvacrol (CAR), and thymol (THY), hereinafter collectively referred to as EOs.^[^ ^9^, ^10^ ^]^ The two linkers contained in PUM168 (PUM168@EOs) are the dianion 4,4′‐biphenyl‐dicarboxylate and the neutral bis‐pyridine‐bis‐amide pillar L1 (Figure 1, left).

These linkers connect paddle‐wheel (SBUs, inset in Figure 1), giving rise to a threefold interpenetrated structure whose asymmetric unit has the formula [Zn_3_(L1)_1.5_(BDC)_3_](DMF)_x_ (BDC = 4,4′‐biphenyl‐di‐carboxylate, DMF = N,N‐dimethylformamide). EOs are recognized as antibacterial agents and find application as botanical pesticides^[^ ^11^, ^12^ ^]^ or active ingredients in food packaging.^[^ ^13^, ^14^ ^]^ Despite the high degree of interpenetration, the framework of PUM168 is highly flexible, as observed during the reversible DMF‐EOs exchanges^[^ ^9^, ^10^ ^]^ and interconversions between different solvates.^[^ ^15^ ^]^ The structural characterization of PUM168 loaded with EOs revealed a higher affinity toward EUG with respect to THY and CAR.^[^ ^10^ ^]^ Moreover, two different types of receptor sites in the framework were recognized, corresponding to the amide group installed on the pillar L1 and the carboxylate group forming the SBU. Notably, EUG shows a strong preference for the SBU, interacting by charge‐assisted H‐bonds, whereas THY and CAR predominantly interact with the amide groups. This preferential guest distribution results in guest‐dependent release profiles, where EUG is released more slowly than THY and CAR. The PUM library includes other pillared MOFs deriving from the combination of the pillar L1 with different dicarboxylic acids, corresponding to structurally diverse frameworks.^[^ ^16^, ^17^ ^]^ Aimed at studying the inclusion and release capacity of other PUM frameworks featured by different entanglement motifs, we focused our attention on PUM210.^[^ ^18^ ^]^ This material is a pillared MOF containing 2,6‐naphthalene dicarboxylate dianions instead of 4,4′‐biphenyldicarboxylate dianions. Its asymmetric unit has the formula [Zn_4_(L1)_1.5_(NDC)_4_(DMF)] (NDC = 2,6‐naphthalene dicarboxylate). Although the chemical components are similar to that of PUM168, several structural features make the two MOFs different. The framework of PUM210 is featured by a fourfold polycatenation of parallel 2D planes (Figure 1, right, Figure 11S), with rectangular microporous channels having a cross section of 7.1 × 11.5 Å^2^ and a potential void volume of 32% of the unit cell (after removal of the residual electron density), corresponding to about 6724 Å^3^ (Figure 11S). The three cubic interpenetrated frames of PUM168 generate instead meandered channels having variable diameters comprised between 15.1 Å and 7.1 Å and a higher void volume, corresponding to 49.7% of the unit cell (after removal of the residual electron density), equivalent to about 2914 Å^3^ (Figure 11S). Moreover, the framework of PUM168 contains exclusively complete paddle‐wheel SBUs, whereas PUM210 contains two different SBUs corresponding to a complete and a truncated paddle‐wheel (Figure 1). In the truncated paddle wheel, a solvent molecule replaces a pyridine of L1, which should enhance the uptake capability of the MOF based on the expected solvent lability. PUM210 also demonstrates lower flexibility than PUM168, as demonstrated by the retention of its structure after several manipulations.^[^ ^19^ ^]^

MOFs have gained increased attention in active food packaging, exploiting their exceptional porosity and tunable surface chemistry to facilitate the encapsulation and controlled release of EOs. These compounds exhibit antioxidant, bacteriostatic, and antibacterial properties, making them highly attractive for the development of active food packaging systems.^[^ ^20^ ^]^ However, the high volatility and liquid form pose a serious challenge: different encapsulating materials and controlled‐release mechanisms have been suggested to provide a controlled release of EOs while maintaining their bioavailability and stability.^[^ ^21^ ^]^ Although the potential toxicity of MOFs must be seriously evaluated, their application in the food packaging field is becoming increasingly prevalent, owing to their nonreactive behavior, and MOFs are now considered very promising encapsulating materials for the development of smart packaging solutions.^[^ ^22^ ^]^

Based on these premises, this study compares the loading capacity of the two MOFs for an equimolar mixture of THY and CAR. Subsequently, we evaluated the thermally controlled release profiles of the two EOs from the two different materials. The results allow for the evaluation of the relative importance of the framework dynamicity and the presence of coordinative unsaturations on the uptake ability of the two microporous pillared MOFs. Regarding the two EOs, they were selected according to their antibacterial activity toward a series of bacterial strains responsible for food spoilage. In our previous study, we demonstrated THY and CAR exhibited distinct antimicrobial activities against Gram‐negative bacteria, namely *Escherichia coli* and *Salmonella Typhimurium*, and Gram‐positive bacteria, namely *Staphylococcus aureus* and methicillin‐resistant *S. aureus*. Specifically, THY showed a predominantly bactericidal effect across all tested strains, while CAR demonstrated a dual action—bactericidal activity against Gram‐negative bacteria and a bacteriostatic effect against Gram‐positive ones. These results underscore the necessity of combining multiple active compounds to achieve broad‐spectrum inhibition of diverse microbial populations.^[^ ^23^ ^]^ The relative amounts of the included guests were evaluated by proton nuclear magnetic resonance (^1^H NMR) spectroscopy and thermal gravimetric analysis (TGA) analysis, and efforts were made to determine the positioning of the guest molecules inside the MOF framework by single‐crystal X‐ray structural characterizations. The thermally induced time‐dependent release profiles from the crystalline materials were monitored by headspace gas chromatography‐mass spectrometry (HS‐GC‐MS) analysis to explore the correlation between the structural features and the release profile of the two MOFs.

## 2 Experimental Section

### 2.1 Materials and Methods

PUM168^[^ ^9^ ^]^ and PUM210^[^ ^18^ ^]^ were synthesized as already reported. For their synthesis, Zn(NO_3_)_2_⋅6H_2_O, 4,4′‐biphenyldicarboxylic acid, 2,6‐naphthalenedicarboxylic acid, and N,N‐dimethylfromamide were commercially available and were used as received. The pillar linker L1 was synthesized as previously reported.^[^ ^24^ ^] 1^H NMR spectra were recorded using a Bruker AV‐400 MHz spectrometer and processed with MestReNova^[^ ^25^ ^]^ software. Chemical shift values are reported in ppm relative to the residual solvent signals of deuterated dimethylsulfoxide (DMSO‐d_6_, δH = 2.50), while coupling constants (J) are expressed in Hz (see Supporting Information, Figures 1S–2S, 5S–8S). The spectra were recorded after 3 and 7 days of soaking. The crystals were digested in a drop of deuterated trifluoroacetic acid (CF_3_COOD, TFA‐d) diluted with DMSO‐d_6_. Thermogravimetric analyses were performed using a PerkinElmer TGA 8000 thermogravimetric analyzer, equipped with a platinum crucible, in a nonreducing atmosphere (air) with a flow ratio of 10 °C/min. Traces were recorded in the interval 30–500 °C (see Supporting Information, Figures 3S–4S). A Quanta FEG 250 (Thermo Fisher Scientific) Scanning Electron Microscope (SEM) equipped with a Bruker XFlash 6 | 30 detector was used for the morphological characterization of the sorbent (see Supporting Information Figures 9S–10S).

### 2.2 X‐ray Crystallography

The diffracted intensities of selected MOF crystals were recorded using a Bruker D8 Venture diffractometer equipped with a kappa goniometer, an Oxford Cryostream, and a Photon II detector, employing microfocused Cu Kα radiation (*λ* = 1.54178 Å). Lorentz polarization and absorption corrections were applied to all experiments. Data reduction was performed using APEX v4 software. The structures were solved using direct methods with SHELXT^[^ ^26^ ^]^ and refined by full‐matrix least squares on all F^2^ values using SHELXL,^[^ ^27^ ^]^ as implemented in Olex2,^[^ ^28^ ^]^ applying anisotropic thermal displacement parameters for all nonhydrogen atoms. The quantification of the residual electron density accounted for disordered solvents/guests was performed after SQUEEZE/PLATON cycles.^[^ ^29^ ^]^ To prevent solvent loss, the crystals were immersed in a drop of Fomblin oil prior to X‐ray diffraction analysis. Experimental crystallographic details are collected in Table 3S. Deposition number 2421352 (for PUM210@*olomix*) contains the supplementary crystallographic data for this paper. These data are provided free of charge by the joint Crystallographic Data Centre and Fachinformationszentrum Karlsruhe http://www.ccdc.cam.ac.uk/structures Access Structure Service.

### 2.3 Soaking of MOFs in Olomix Mixture

The *olomix* mixture containing an equimolar ratio of CAR and THY was prepared by dissolving 1.952 g of THY (0.013 mol) in 2 mL of CAR (0.013 mol, d = 0.976 g/mL at 20 °C). The mixture was directly used in soaking experiments.

Crystals of pristine MOF of comparable dimensions (either PUM168 or PUM210, approximately 20 mg) were introduced in a vial and suspended in 2 mL of acetone. The vial was then placed on a rotating plate for 2 hours at room temperature. The supernatant was replaced with fresh acetone every 30 minutes. The occurred solvent exchange was determined by ^1^H NMR spectroscopy.

MOF@ACE crystals (20 mg) were introduced in a vial and suspended in 2 mL of *olomix*. The vial was placed on a rotating plate at room temperature for 20 minutes. Then, the supernatant was replaced with a fresh aliquot (2 mL) of *olomix* and left on the rotating plate for an additional 24 hours. The different experiments were conducted, maintaining constant the ratio between the mass of crystals and the volume of the binary mixture. Then, the *olomix* uptake was carried out at 25 °C, monitoring the progress by ^1^H NMR spectroscopy after 3 and 7 days. To facilitate the comparison of the uptake data, the integration areas of the guest signals were compared with that of L1 on the basis of the MOF framework formula, [Zn_2_(L_1_)(BPDC)_2_] and [Zn_2_(L1)_0.75_(NDC)_2_(DMF)_0.5_], respectively. The longest time interval was chosen to ensure equilibration on the basis of the previous results found in the uptake of EO_s_ by PUM168.^[^ ^9^, ^10^ ^]^ To investigate possible temperature effects, the soaking experiments were repeated at 40 °C with the crystals immersed in *olomix* for 3 days. After this period, the crystals were analyzed by ^1^H NMR spectroscopy and TGA analysis.

### 2.4 Headspace Gas‐Chromatography Mass‐Spectrometry

The release profiles of THY and CAR from PUM168@*olomix* and PUM210@*olomix* were evaluated over time operating at room temperature by means of headspace gas chromatography coupled with mass spectrometry (HS‐GC‐MS). The release profiles were compared to those of pure EOs. The following procedure was applied: 1 mg of each material was introduced into a 10 mL glass vial and maintained uncapped under a laminar flow hood at room temperature (25.2 ± 0.5 °C; *n* = 3, independent measurements per day) for 0, 3, 7, 10, and 15 days, respectively. After the scheduled release time had expired, the vials were sealed with PTFE/silicone septa, equilibrated at 30 °C for 5 minutes, and submitted to HS‐GC‐MS analysis. 1 mL of the headspace above the sample was introduced into the gas chromatograph injection port by means of a PAL COMBI‐xt autosampler (CTC Analytics AG). An HP 6890 Series Plus gas chromatograph equipped with an MSD 5937 mass spectrometer (Agilent Technologies) was used for GC‐MS analyses. Separation was carried out on an Rxi‐624Sil MS column (length: 30 m, internal diameter: 0.25 mm, film thickness: 1.4 µm) (Restek) using the following temperature program: 100 °C, 10 °C/min to 160 °C, 15 °C/min to 250 °C. Helium was used as a carrier gas at a constant flow rate of 1 mL min^−1^. The injector was held at 250 °C, operating in split mode (split ratio: 5:1). The transfer line and source were maintained at the temperatures of 270 and 150 °C, respectively. Full scan electron ionization (EI) data were acquired under the following conditions: ionization energy: 70 eV; mass range: 40−200 amu; scan time: 3 scan/s; electron multiplier voltage: 2000 V. A solvent delay of 2 minutes was applied. Signal acquisition and data handling were performed using the HP ChemStation software (Agilent Technologies).

## 3 Results and Discussion

### 3.1 Activation protocol for PUM168 and PUM210

**Figure 2:**
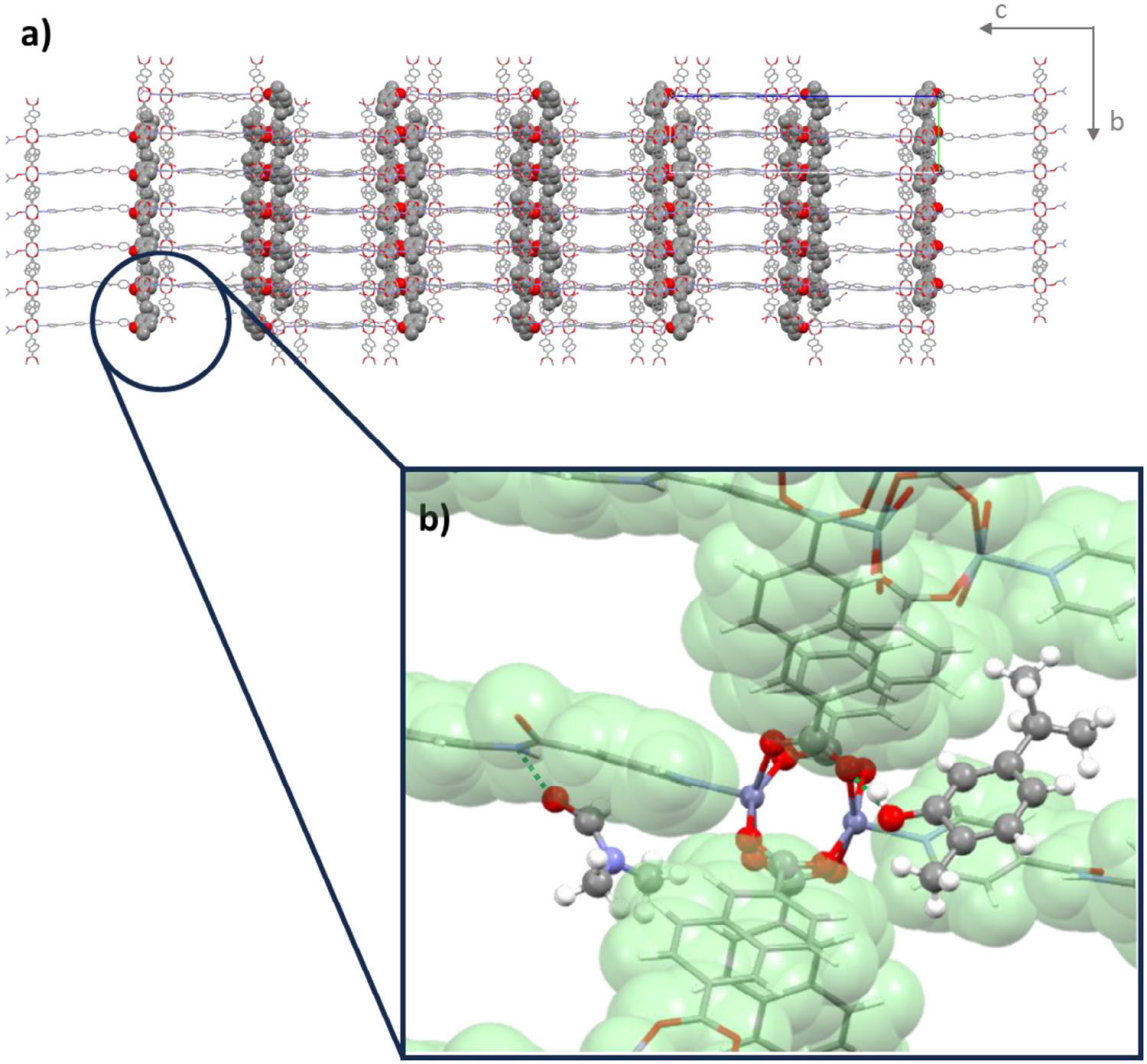
a) Representation of the polycatenated structure of PUM210@*olomix*, highlighting the packing of CAR molecules (in space‐fill view). b) Insight into the interaction between the CAR molecule and the paddle wheel in PUM210, in the background the residual hydrogen‐bonded molecule of DMF.

In a preliminary step, the crystals of PUM168 and PUM210 were treated to replace the solvent used in the synthesis with a suitable one. Since PUM168 and PUM210 derive from solvothermal syntheses conducted in DMF, the voids present in the pristine crystals are filled with DMF, a high boiling solvent not compatible with the intended application as antibacterial materials, due to its toxicity^[^ ^30^, ^31^ ^]^ and suspected carcinogenicity.^[^ ^32^ ^]^ Moreover, the high hydrogen‐bond propensity of DMF makes this solvent highly competitive for the uptake of EOs, especially for binding to the amide groups.^[^ ^9^, ^18^ ^]^ The DMF removal by thermal treatment under vacuum was discharged to avoid any crystal damage,^[^ ^33^ ^]^ preferring a milder solvent exchange protocol (See Experimental section for details).^[^ ^18^, ^34^ ^]^ This involves the exchange of DMF with a more volatile and less toxic solvent, such as acetone (ACE), compatible, for instance, with food packaging materials.^[^ ^35^ ^]^ The DMF‐to‐ACE replacement was carried out by soaking the pristine crystals in pure acetone and it was easily monitored by ^1^H NMR spectroscopy (Figure 1S) after digestion of the crystals in a mixture of TFA‐d and DMSO‐d_6_. The resulting material, PUM168@ACE, was obtained within 2 hours of soaking. As reported in previous structural analysis,^[^ ^9^ ^]^ the DMF‐to‐ACE exchange provokes a sliding of the interpenetrated frames which reduces the potential void from 49.5% (PUM168) to 23.2% (PUM168@ACE) (Figure 12S). This structural rearrangement preserves the framework connectivity and entanglement. It is worth mentioning that the reduction in the void volume does not immediately translate into the loss of the inclusion capacity of the MOF. In fact, contact with a suitable guest may restore sufficient porosity for guest entering, as observed during both the reversible interconversion of PUM168@ACE into PUM168^[^ ^9^, ^10^ ^]^ and the repeated solvent exchanges involving different PUM168 solvates with different potential voids.^[^ ^15^ ^]^ In the case of PUM210, the framework is substantially rigid, remaining intact after several manipulations, such as transmetallation^[^ ^18^ ^]^ and activation.^[^ ^19^ ^]^ The DMF exchange resulted almost complete within five days of soaking (Figure 2S). The residual trace of the pristine solvent, counting for one and a half molecule per asymmetric unit, was mainly attributed to DMF coordinated to the truncated paddle wheel. With both materials, prolonged soakings did not alter the NMR profile, but resulted in a severe crystal fragmentation. This markedly different framework flexibility is expected to impact the uptake capacity of the two MOFs.

### 3.2 THY and CAR uptake

**Scheme 1:**
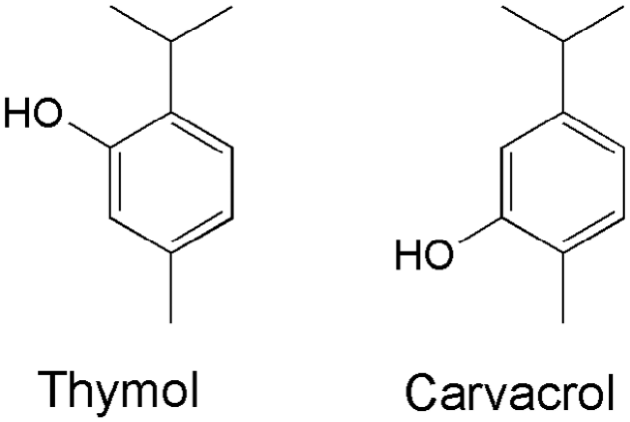
Molecular structures of THY and CAR.

The molecular structures of the two active EOs THY and CAR are depicted in Scheme 1.

**Figure 3:**
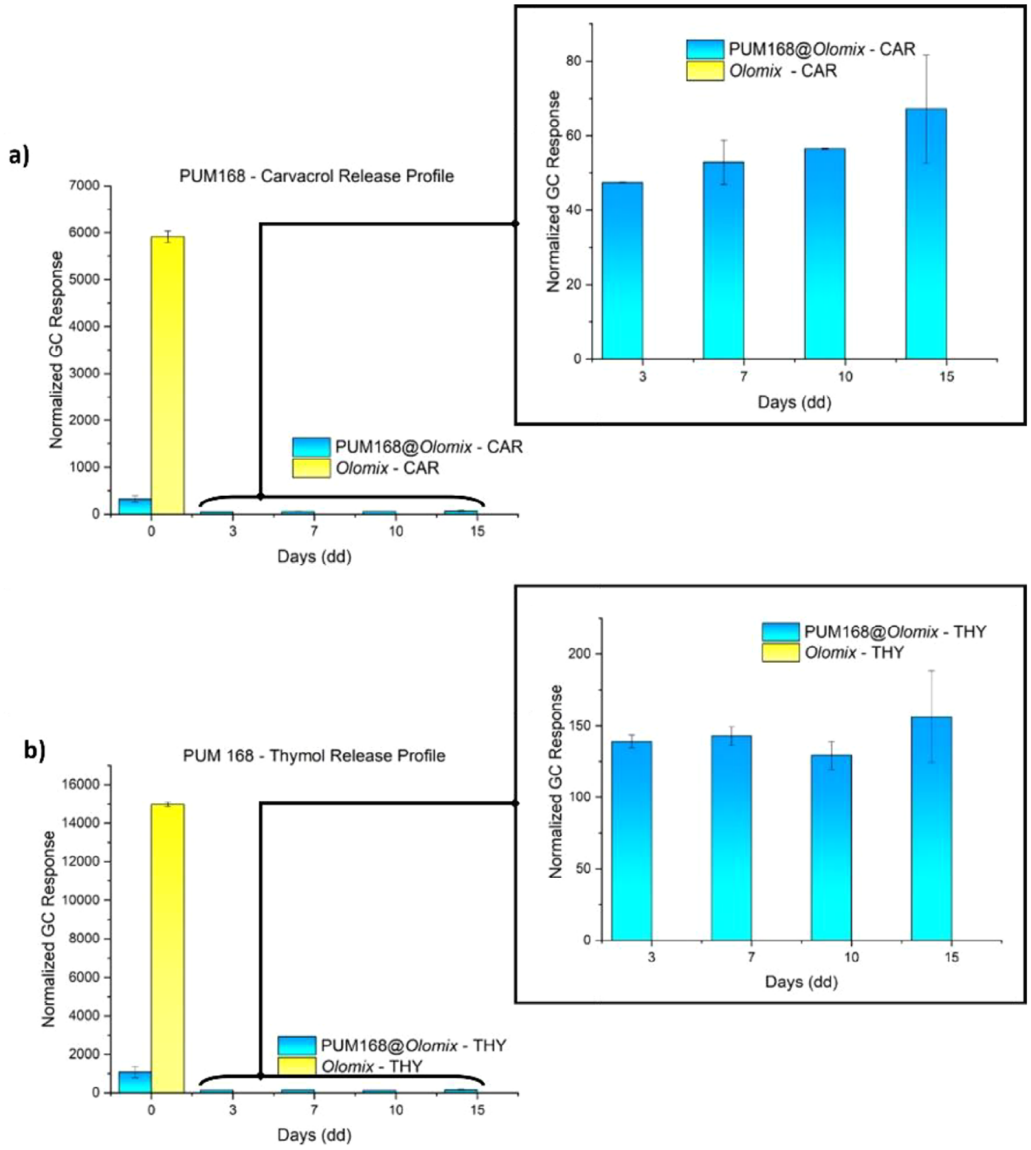
EO release profiles expressed as normalized GC response (a.u.) of a) CAR and b) THY. The results obtained from PUM168@*olomix* crystals (blue bars) and pure *olomix* (yellow bars) are shown as a function of time.

THY and CAR are positional isomers characterized by distinct physical properties. In particular, THY is solid at room temperature, while CAR exists as a liquid. Consequently, an equimolar binary mixture was created by dissolving THY in CAR, and the mixture, hereinafter referred to as *olomix*, was used as soaking medium for the crystals of PUM168@ACE and PUM210@ACE (see the Experimental section for experimental details). The molecular volumes of the two molecules are similar, being 166 Å^3^ and 161 Å^3^ for THY and CAR, respectively.^[^ ^36^ ^]^ The estimation of the relative amounts of included EOs was determined via ^1^H NMR analysis. To reduce errors deriving from guest molecules physically adsorbed on the surface of the crystals, these were carefully passed over a paper filter before being subjected to NMR analysis. The number of included guest molecules reported in Table 1 is referred to congruent portions of the framework of the two MOFs, corresponding to [Zn_2_(L1)(BDC)_2_] and [Zn_2_(L1)_0.75_(NDC)_2_(DMF)_0.5_] for PUM168 and PUM210, respectively. A comparison of the hosting capacity of the two pillared MOFs reveals that the interpenetrated frameworks exhibit a greater uptake ability than the polycatenated one, regardless of the temperature. As can be seen in Table 1 (entry 1, 3 days), at 25 °C and after 3 days of soaking, PUM168@ACE includes 4 molecules of guest, with CAR (3 molecules, 28% mass) preferred over THY (1 molecule, 9% mass). Under the same conditions, PUM210@ACE includes only 0.5 molecules of each guest, corresponding to an uptake of about 12% in mass (entry 2, 3 days). Prolonging the soaking to 7 days does not significantly affect the total amount of guest molecules included. In fact, in the case of PUM168@ACE, the additional entering of only one molecule of THY (entry 1, 7 days) is observed, whereas in the case of PUM210@ACE the entering of an additional half molecule of CAR (entry 2, 7 days) is obtained. These data indicate that equilibrium is reached within 3 days of soaking. The same equilibrium is reached at 40 °C, as can be inferred from entries 3 and 4 of Table 1. Higher temperatures were not tested to avoid excessive crystal degradation. TGA analyses carried out on crystals soaked in *olomix* for 3 days at 40 °C showed weight loss percentages in good agreement with the expected data. In the case of PUM168@ACE, a weight loss of 33% was obtained compared to the expected 37% in the temperature range of 30–235 °C. In the same temperature interval, the weight loss percentage observed in the case of PUM210@ACE was 25%, compared to the expected value of 18% (Figure 3S). In this case, the discrepancy may be attributed to the retained molecules of solvents, especially DMF, as reported below. In both cases, the framework decomposition started at about 400 °C, a temperature similar to that observed for the pristine materials. The data collected in Table 1 indicate that the flexible interpenetrated PUM168 has higher hosting capacity than the rigid polycatenated PUM210. This difference cannot be solely attributed to their different potential void volumes, which favor PUM210@ACE, but rather to the presence of the coordinated DMF in the latter and to its greater rigidity. The absence of guest competitors and the pronounced flexibility of the threefold interpenetrated framework of PUM168 allow it to adapt dynamically to changes in its content, ensuring sufficient attractive interactions with the incoming guest molecules throughout the soaking process.

**Table 1:** ^1^H NMR estimation of the guest uptake for PUM168@ACE and PUM210@ACE after 3 and 7 days of soaking at 25 or 40 °C.^[a]^

Entry	MOF	Soaking temperature [°C]	Time [days]	Molecules of THY included [mass %]	Molecules of CAR included [mass %]
1	PUM168@ACE	25	3	1 (9)	3 (28)
			7	2 (17)	3 (25)
2	PUM210@ACE	25	3	0.5 (6)	0.5 (6)
			7	0.5 (6)	1 (12)
3	PUM168@ACE	40	3	1 (9)	3 (28)
4	PUM210@ACE	40	3	0.5 (6)	1 (12)

^[a]^ Data are averaged over two different experiments.

Consequently, the spaces required to accommodate CAR and THY molecules are formed during the soaking process, driven by the continuous establishment of supramolecular contacts between the MOF walls and the guest molecules. In contrast, the intrinsic rigidity of PUM210 reduces its adaptability, and the guest molecules can pass through the MOF framework without forming relevant stabilizing interactions. This different behavior is evidenced also by the TGA traces of the two loaded crystalline materials. In the case of PUM168@*olomix*, the TGA profile is indicative of a multistep guest extrusion process, from the continuous framework modification following the guest extrusion. In contrast, the TGA trace for PUM210@*olomix* shows a more constant guest release. The preferential CAR uptake found with PUM168@ACE can be rationalized considering the higher steric congestion experienced by the OH group of THY due to the vicinal *i*‐Pr group. This steric congestion likely hampers the plumbing of THY through the MOF channels, preventing the establishment of stable host‐guest interactions. Moreover, based on previous findings,^[^ ^9^ ^]^ CAR has a stronger tendency than THY to interact with the carboxylates contained in the SBUs, thus forming hydrogen bond contacts. The loading capacities of the two mixed‐ligand MOFs compare well with the ones found with homoleptic MOFs, such as MIL‐100(Fe)^[^ ^37^ ^]^ and UiO‐66‐(COOH)_2_^[^ ^38^ ^]^ whose efficiency, however, was tested only against single guests.

To obtain structural information on the supramolecular organization adopted by the included guest molecules, the loaded crystals were subjected to single‐crystal X‐ray structural analysis.

### 3.3 Structural analysis

Single‐crystal X‐ray diffraction analysis is the only technique able to provide a detailed structural description of the guest distribution within the MOF framework, highlighting the type of intermolecular contacts responsible for its uptake. Therefore, several efforts were devoted to isolate X‐ray quality single crystals of the loaded materials. During the soaking experiments, the pristine crystals of both MOFs exhibited crystal fragmentation. Unfortunately, in the case of PUM168@*olomix*, the damages undergone by the crystals resulted too severe, and they were no longer available for single‐crystal X‐ray analysis. A morphological analysis by SEM revealed a severe cracking of the loaded crystals (Figure 9S), responsible for the loss of crystallinity. The cracking is imputable to the excessive mechanical stress undergone by the framework during guest trafficking, and it was already observed during soaking of PUM168 in pure CAR.^[^ ^39^ ^]^ However, the PXRD trace of PUM168@*olomix* is similar to that of the starting material, indicating a substantial retention of framework structure. In the case of PUM210@*olomix*, the crystals maintained their integrity, as evidenced by morphological analysis via SEM (Figure 10S) making them suitable for single‐crystal X‐ray analysis. It is worth noting that PUM210@*olomix* presents the same perturbation of periodicity already shown by PUM210,^[^ ^18^ ^]^ related to an uncorrelated displacement of the metal nodes along the paddlewheel axis and causing diffuse scattering, which affects the quality of the data. Besides this intrinsic structural disorder, data analysis also reveals a twinning, which further affects the modeling of intensities, resulting in a quite high R‐factor, which, however, does not prevent an exhaustive structural characterization of this MOF. Figure 14S reports an extensive analysis of the above two factors affecting the refinement. The structure evidences an asymmetric unit consisting of four Zn ions, four dicarboxylate dianions, one and a half molecules of L1, one molecule of CAR (100% occupancy), and one and a half molecules of DMF (Figure 13S). One of the DMF molecules is hydrogen‐bonded to an amide group (50% occupancy, N─H…O═C = 2.87 Å), whereas the other is coordinated to one Zn belonging to the truncated paddle‐wheel (100% occupancy, Figure 2). The residual electron density accounts for 74 electrons, located in a volume of 716 Å^3^ of the asymmetric unit: this value is in accordance with the presence of a disordered molecule of a phenolic guest probably located in the mono‐dimensional channel running along the crystallographic axis *a*. Based on the ^1^H NMR characterization, we assume that the unmodeled guest corresponds to a THY molecule, for a whole MOF formula corresponding to [Zn_4_(L1)_1.5_(NDC)_4_(CAR)(DMF)_1.5_]⋅(THY). The MOF framework is very similar to that of pristine PUM210, with the same type of catenation and SBUs, a further confirmation of the rigidity of the polycatenated framework (Figure 2). For a comparison of the crystal packing of PUM210 and PUM210@*olomix*, see Figures 15S and 16S.

The structure determination reveals that the incoming phenolic guests are not able to remove the coordinated DMF molecule, which remains bound to Zn in the truncated paddle wheel (Figure 2a). The included molecule of CAR confirms its preference to interact with the carboxylate group of a complete paddle‐wheel unit (O‐H…OOC = 2.84 Å) through a hydrogen bond (Figure 2b), as observed crystallographically in a series of structures of PUM168@EOs.^[^ ^10^, ^39^ ^]^ The CAR molecules are trapped in a narrow pocket generated by the dicarboxylate planes and located in close proximity to the coordinated molecule of DMF, rather than being distributed along the 1D channels. The disposition of the CAR molecules follows a twofold screw axis running along the crystallographic axis *b* (Figure 2a). The relative orientation of the carbonyl amide groups of L1 is different from that found in the pristine PUM210. In the pristine crystal, the C═O groups are always staggered in a *transoidal* arrangement with a twist angle of about 150° (Figure 17S). In PUM210@*olomix*, this arrangement is obtained only in one out of the three pillars that form a single frame. This is likely the only relevant flexibility involving the polycatenated framework during the soaking, a further confirmation of its intrinsic rigidity. Nicely, the PXRD trace of PUM210@*olomix* is comparable to that calculated from the single crystal structure, indicating that the solved structure is representative of the bulk material (Figures 19S and 20S).

### 3.4 HS‐GC‐MS release of THY and CAR from PUM168 and PUM210

The possibility of using MOF crystals loaded with active natural components opens the way to their use as functional materials, for instance, to produce active food packaging,^[^ ^1^, ^22^ ^]^ where the slow release of antibacterial agents is expected to increase the shelf life of the packaged product.^[^ ^40^ ^]^ To test the potential of the two crystalline loaded materials, the room temperature release profiles of THY and CAR from PUM168@*olomix* and PUM210@*olomix* were monitored over time by HS‐GC‐MS. Compared to *olomix*, PUM168@*olomix* shows a much more gradual and steady release extending for at least 15 days (Figure 3, Tables 1S). In contrast, a markedly different behavior is observed with *olomix*, whose release profile disappears within 24 hours.

**Figure 4:**
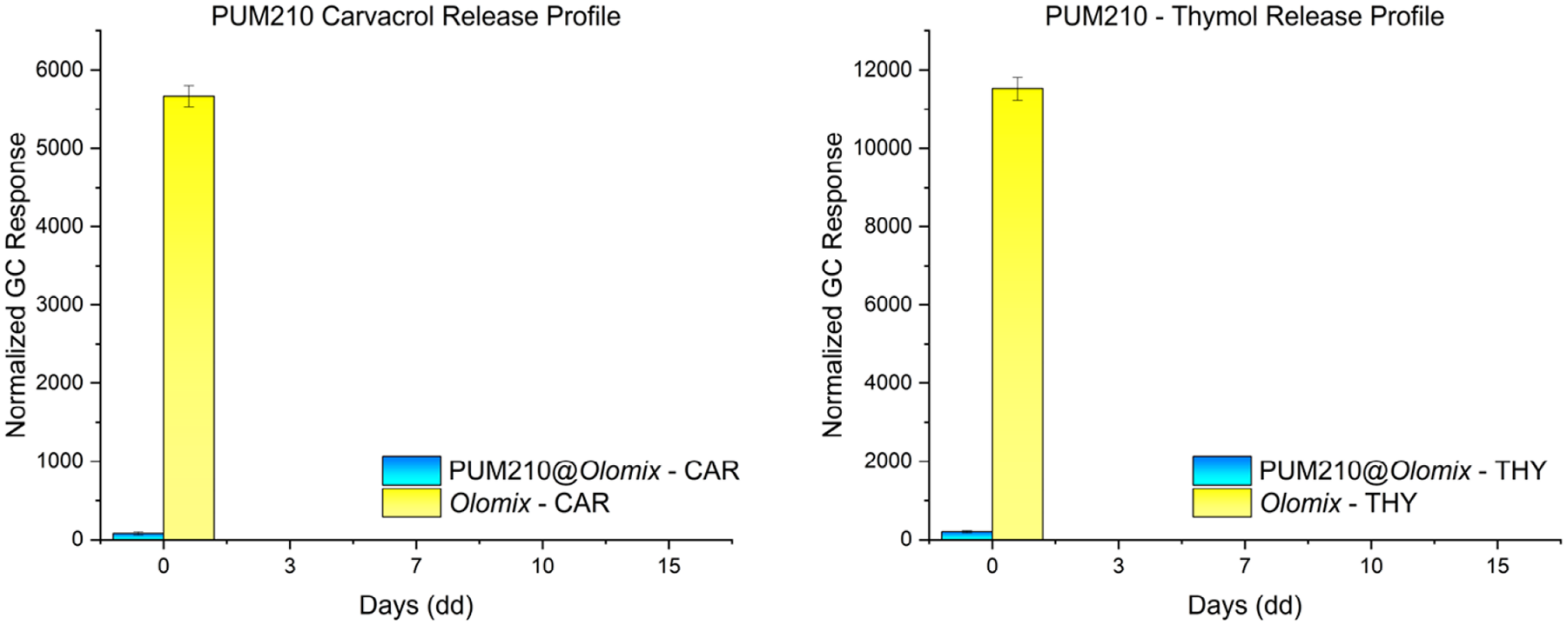
EO release profiles expressed as normalized GC response (a.u.) of: a) CAR and b) THY. The results obtained from PUM210@*olomix* crystals (blue bars) and pure *olomix* (yellow bars) are shown as a function of time.

Interestingly, THY is released preferentially compared to CAR, even though the latter is more volatile (3.09 Pa vs. 2.20 Pa at 25 °C)^[^ ^41^, ^42^ ^]^ and present in larger amounts inside the crystals. This behavior can be explained on the basis of the different positioning of the two guests in the crystalline container.^[^ ^9^ ^]^ CAR is expected to mainly interact with the carboxylates in the SBU through polarizing‐assisted hydrogen bonds, a stronger interaction than that involving THY and the amide functionality of the pillar. Moreover, considering the different flexibility of the two frameworks, the adaptive behavior of PUM168 ensures continuous interactions between the guest molecules not yet extruded and the MOF walls, leading to a slow release. In contrast, the release profiles of PUM210@*olomix* are comparable to those of *olomix* (Figure 4, Table 2S), showing a massive and almost complete guest release within the first 24 hours, indicating the formation of relatively weak host‐guest interactions. In addition, CAR is released in larger amounts than THY, in accordance with the trend of the vapor pressure values and with the calculated loading percentages. In this context, the scarce flexibility of the framework prevents the stabilization of host‐guest interactions during the guest extrusion, which is released mainly on the basis of its volatility.

## 4 Conclusion

This work demonstrates that the microporous Zn‐based MOFs investigated are nice candidates to construct multicomponent systems  for the controlled release of active compounds. In this study, it was demonstrated that the two pillared MOFs PUM168@ACE and PUM210@ACE exhibit good uptake ability toward an equimolar binary mixture of CAR and THY, with PUM168@ACE showing the higher loading capacity. This was rationalized on the basis of the higher flexibility of PUM168 compared to PUM210 as well as the presence, in the latter, of residual coordinated DMF molecules. A preferential inclusion of CAR over THY is always obtained; this behavior can be related to the different steric congestion around the hydroxyl group of the guest. This group is indeed involved in the intermolecular contacts responsible for the stable positioning of the guest molecule inside the MOF framework,^[^ ^9^, ^10^, ^39^ ^]^ as highlighted by the structural analysis conducted on crystals of PUM210@*olomix*.This investigation highlighted the presence of a molecule of CAR hydrogen bonded with a carboxylate group of the SBU. Although not modeled, THY is expected to interact mainly with the amide group through weaker hydrogen bonds. The HS‐GC‐MS monitoring of the guest release from the two MOFs gives clear evidence of the superior ability of PUM168@*olomix* to release the active volatile guests in a controlled and prolonged way. The different release profiles are likely due to the combination of two distinct effects: the different guest positioning within the MOF framework and the different flexibility of the material. The stronger interactions involving CAR and the higher adaptability of the threefold interpenetrated framework of PUM168 assure the intermolecular contacts necessary to prolong the guest release up to 15 days at room temperature. These findings pave the way for the development of functional materials that can be applied in fields requiring the controlled release of antibacterial agents. This topic is currently under investigation in our laboratory.

## Supporting Information

The data supporting the finding are available in the Supplementary Material.

## Conflict of Interests

The authors declare no conflict of interest.

## Supporting information



Supporting Information

## Data Availability

The data that support the findings of this study are available in the supplementary material of this article.
