# Supplementary material for: Effect of the Entanglement of Microporous Pillared MOFs on the Uptake and Release Profiles of Essential Oil Components
Source: Chemistry. 2025 Jun 4;31(38):e202501167. doi: 10.1002/chem.202501167 (PMC12238909; doi:10.1002/chem.202501167)
Supplement: Supplementary file 1 — Supporting Information [file CHEM-31-e202501167-s001.docx]

**Supporting information**

**Effect of the Entanglement of Microporous Pillared MOFs on the Uptake and Release Profiles of Essential Oil Components**

Dario Giovanardi,^[a,b]^ Erika Ribezzi,^[a]^ Marta Napolitano,^[a]^ Martina Orlandini,^[a]^ Nicolò Riboni,^[a,c]^ Paolo Pio Mazzeo,^[a,~~c~~d]^ Alessia Bacchi,^[a,b,~~c~~d]^ Federica Bianchi,^[a,c]^ Maria Careri,^[a]^ Paolo Pelagatti^[a,b]^*

[a] Dr. D. Giovanardi, E. Ribezzi, M. Napolitano, Dr. M. Orlandini, Dr. N. Riboni, P.P. Mazzeo, Prof. A. Bacchi, Prof. F. Bianchi, Prof. M. Careri, Prof. P. Pelagatti
Department of Chemical Sciences, Live Science and Environmental Sustainability
University of Parma
Parco Area delle Scienze 17/A, 43124, Parma, Italy
E-mail: [paolo.pelagatti@unipr.it](mailto:paolo.pelagatti@unipr.it)

[b] Dr. D. Giovanardi, Prof. A. Bacchi, Prof. P. Pelagatti
Interuniversity Consortium of Chemical Reactivity and Catalysis (CIRCC)
Via Celso Ulpiani 27, 70126 Bari, Italy

[c] Dr. N. Riboni, Prof. F. Bianchi

Interdepartmental Center for Packaging

Tecnopolo, Padiglione 33, Campus Universitario, 43124 Parma, Italy

[d] ~~Prof~~ Dr. P.P. Mazzeo, Prof. A. Bacchi
Biopharmanet-tec
Parco Area delle Scienze 27/A, 43124, Parma, Italy

Supporting information for this article is given via a link at the end of the document.

**Summary**

[**^1^H NMR Spectroscopy** 2](#_Toc196929331)

[**^1^H NMR spectra of PUM168 and PUM210 after the activation protocol** 2](#_Toc196929332)

[**General procedure for the calculation of DMF molecules from ^1^H NMR spectra** 3](#_Toc196929333)

[***OLOMIX* SOAKING EXPERIMENT** 3](#_Toc196929334)

[**General procedure for the calculation of *olomix* molecules from ^1^H NMR spectra** 3](#_Toc196929335)

[**TGA obtained from the soaking of PUM168 and PUM210 in *olomix* solution** 4](#_Toc196929336)

[**^1^H NMR spectra of the guest uptake experiments.** 5](#_Toc196929337)

[**ESEM ANALYSIS** 9](#_Toc196929338)

[**ESEM images of PUM168 after *olomix* soaking experiment** 9](#_Toc196929339)

[**SC-XRD ANALYSIS** 10](#_Toc196929340)

[**SC-XRD analyses on PUM210@*olomix*** 10](#_Toc196929341)

[**PXRD ANALYSIS** 15](#_Toc196929342)

[**HS-GC-MS RELEASE** 17](#_Toc196929343)

[**HS-GC-MS analysis: sample preparation and analytical protocol** 17](#_Toc196929344)

# **^1^H NMR Spectroscopy**

## **^1^H NMR spectra of PUM168 and PUM210 after the activation protocol**


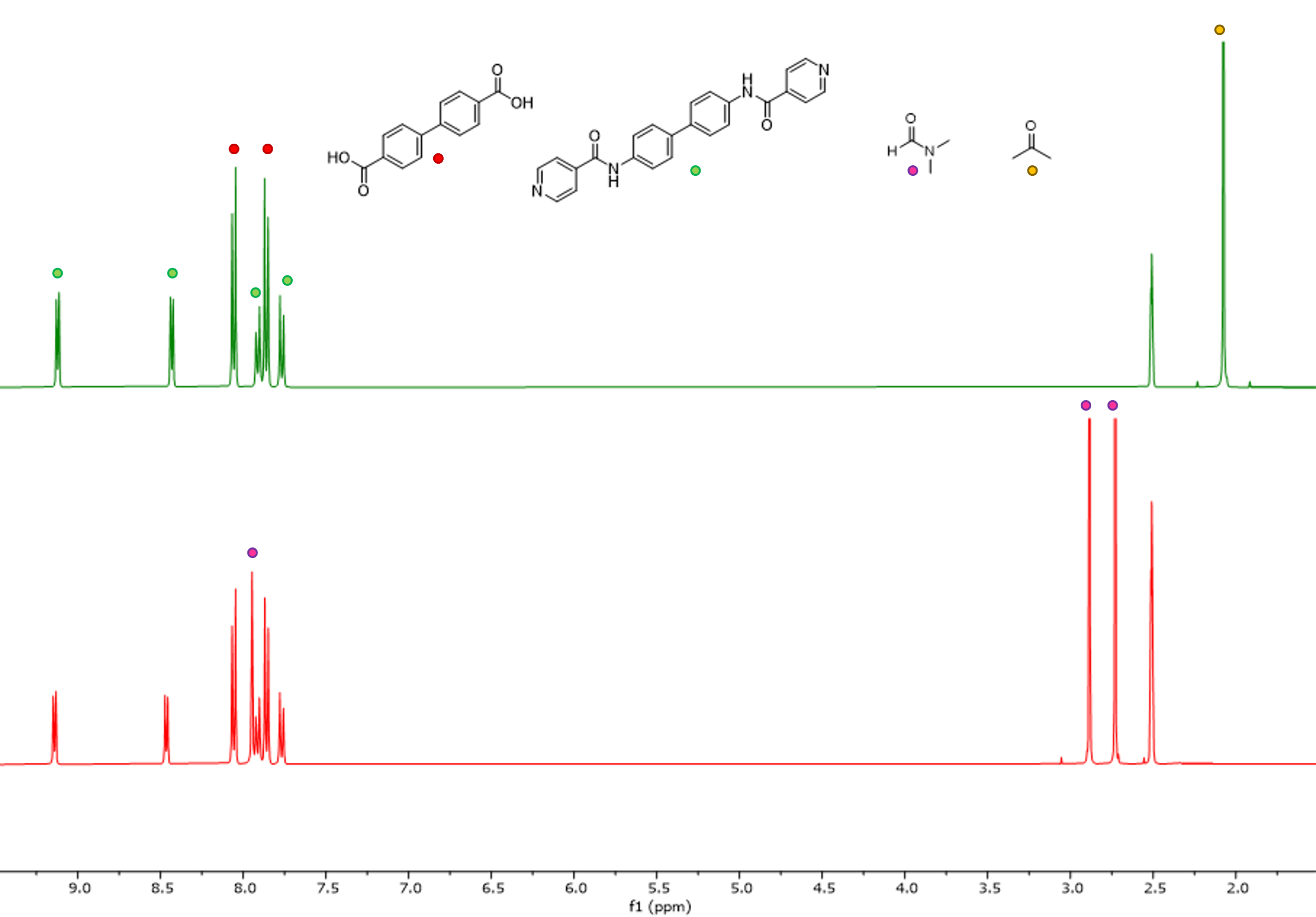


**Figure 1S.** Comparison of ^1^H NMR spectra of **PUM168** as pristine (red spectrum) and after 2h of soaking in acetone (green spectrum), in TFA-d/DMSO-d_6_, 400 MHz, 25°C. The disappearance of the signals of DMF (purple dot) is evident.


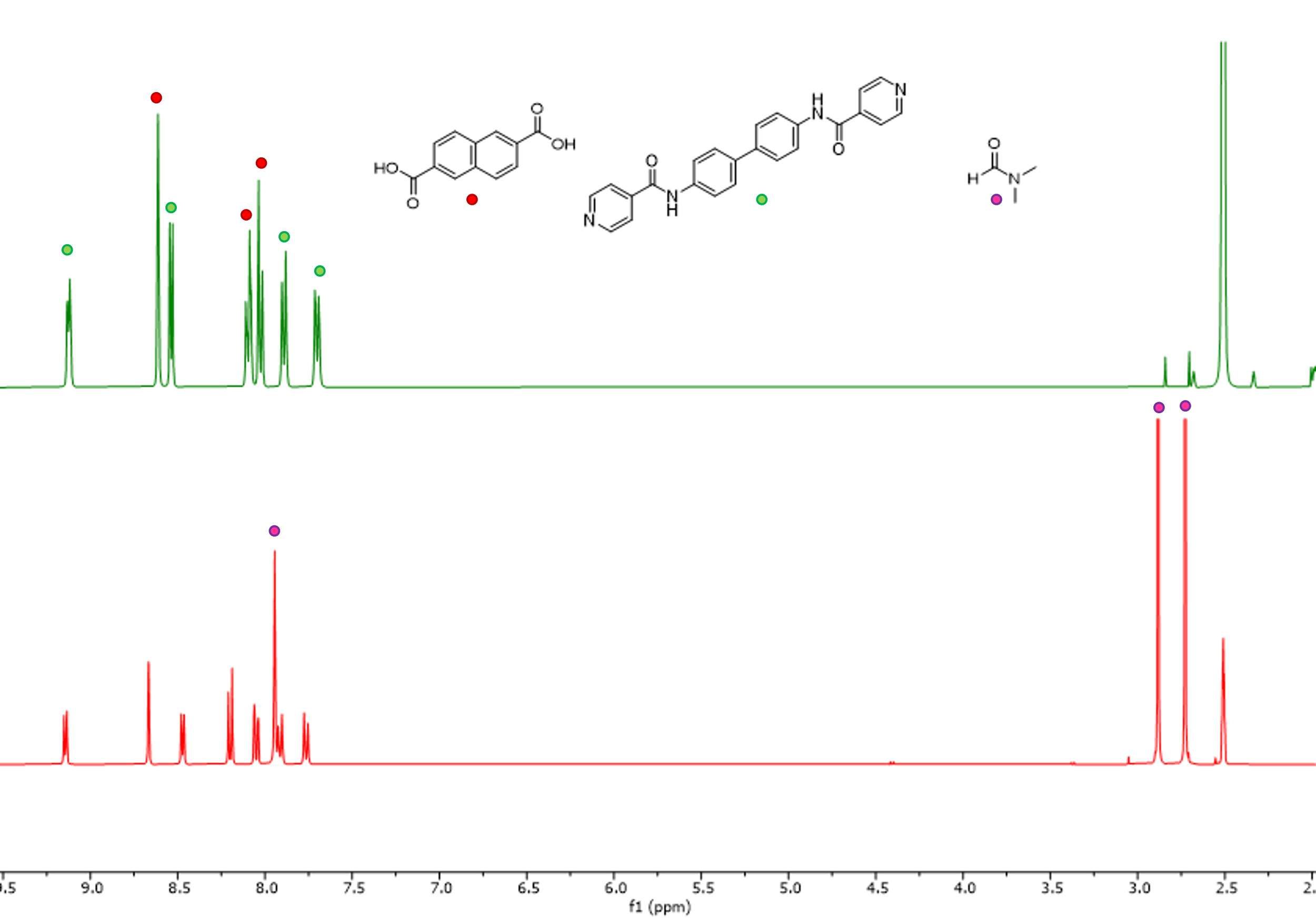


**Figure 2S.** Comparison of ^1^H NMR spectra of **PUM210** as pristine (red spectrum) and after 5 days of *soaking* in acetone (green spectrum), in TFA-d/DMSO-d_6_, 400 MHz, 25°C. The signals referring to residual DMF are indicated by the purple dot in the aliphatic and aromatic regions of the spectrum.

## **General procedure for the calculation of DMF molecules from ^1^H NMR spectra**

From ^1^H NMR analyses performed on the pristine crystals of the MOFs, it is possible to calculate the number of DMF molecules present within the cavities. The calculation is done by considering the asymmetric unit of the different MOFs, which have formula [Zn_4_(L1)_1.5_(NDC)_4_(H_2_O)]_n_·xDMF for PUM210 and, [Zn_3_(L1)_1,5_(BPDC)_3_(DMF)_5_]·xDMF for PUM168.

The integration area of the signal of the pyridine protons in *meta* position with respect to nitrogen were normalized to 6 protons, as found in the asymmetric unit. Consequently, the integration area of one of the CH_3_ singlets of DMF was divided by three. The value obtained corresponds to the number of solvent molecules *per* asymmetric unit.

# ***OLOMIX* SOAKING EXPERIMENT**

## **General procedure for the calculation of *olomix* molecules from ^1^H NMR spectra**

From ^1^H NMR analyses performed on MOF crystals it is possible to calculate not only the number of DMF molecules present within the cavities, as described above, but also the number of the *olomix* components, analyzing the **MOF@*olomix*** crystals. Also in this case, the calculation was done by considering the asymmetric unit of the different MOFs, which have formula [Zn_4_(L1)_1.5_·(NDC)_4_·(DMF)_1.5_]_n_ for PUM210 and, [Zn_3_(L1)_1,5_(BPDC)_3_] for PUM168. Thus, we chose to normalize the integration area of the aromatic signal corresponding to the pyridine protons in *meta* position with respect to nitrogen, following the same approach used for calculating the trapped DMF molecules. This was set to 6 protons for both the MOFs. Consequently, to calculate the number of molecules corresponding to THY and CAR components, we considered the methyl signals from the methyl groups of each compound. So, dividing these values by three, we obtained the number of guest molecules present within the MOF cavities. From the obtained values, it was also possible to calculate the relative mass percentage of the two guests using the following formula: [guest mass/(asymmetric unit mass+guests mass)]x100. Rounding off values by 0.5. The chemical shift values of the chosen proton probes are not affected by the presence of deuterated TFA.

To compare the uptake abilities of the two MOFs, the empirical formula of the two material was normalized by considering the smallest building block of the material. In the case of PUM168 the final empirical formula was multiplied by a scaling factor 2/3 to scale to a Zn_2_ containing subunit. In details the formula is: [Zn_2_(L1)(BPDC)_2_].In the case of PUM210, the scaling factor adopted was ½, resulting in a final formula of [Zn_2_(L1)_0.75_(NDC)_2_(DMF)_0.5_]. The values reported in the Table 1 in the main text refer to the calculated molecules of guest per congruent portions of the two frameworks, corresponding to [Zn_2_(L1)(BDC)_2_] and [Zn_2_(L1)_0.75_(NDC)_2_(DMF)_0.5_] for PUM168 and PUM210, respectively.

## **TGA obtained from the soaking of PUM168 and PUM210 in *olomix* solution**


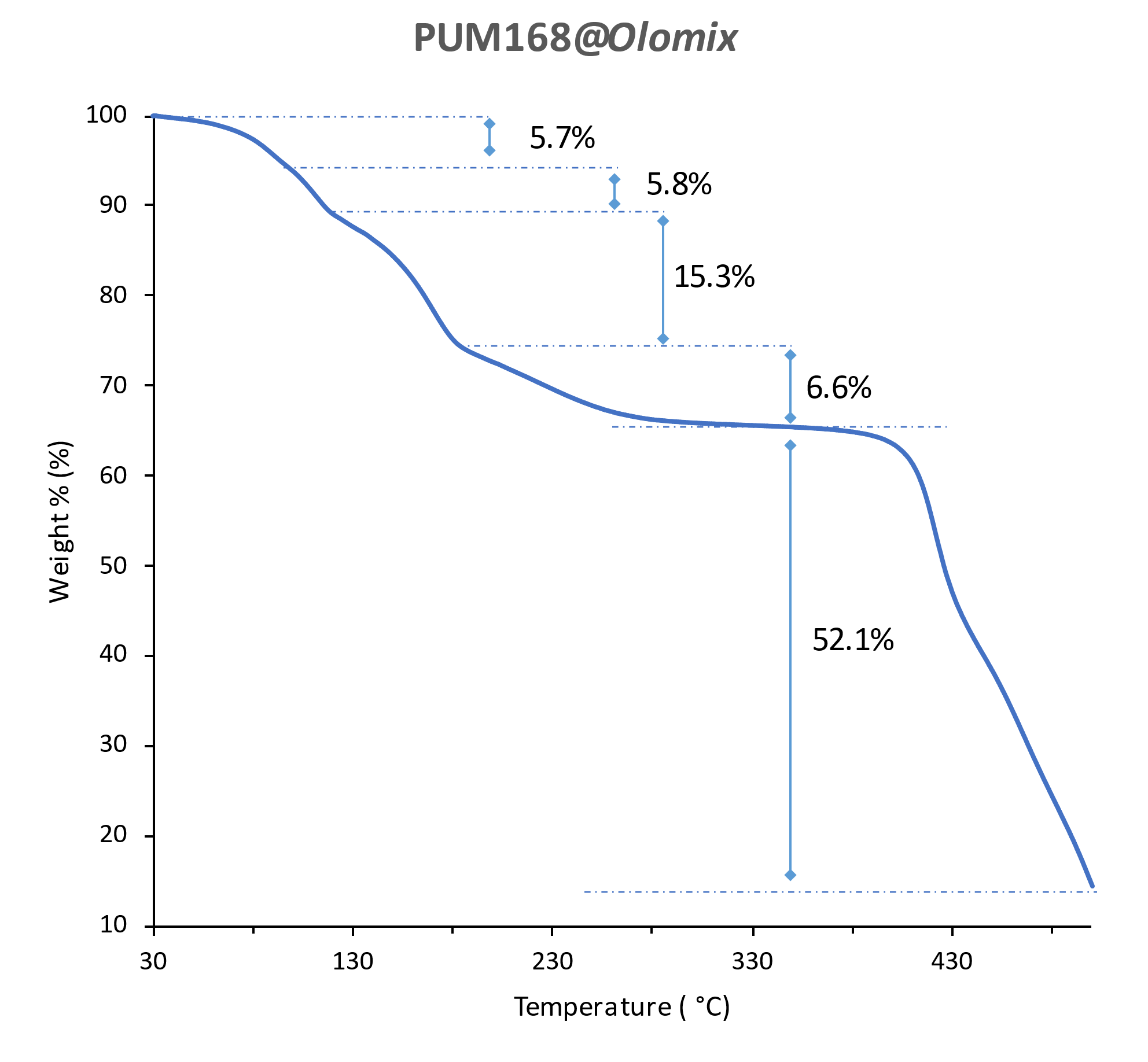


**Figure 3S.** TGA of **PUM168@*olomix*** after three days of soaking in the equimolar mixture, air, 10°C/min, 30-500 °C.


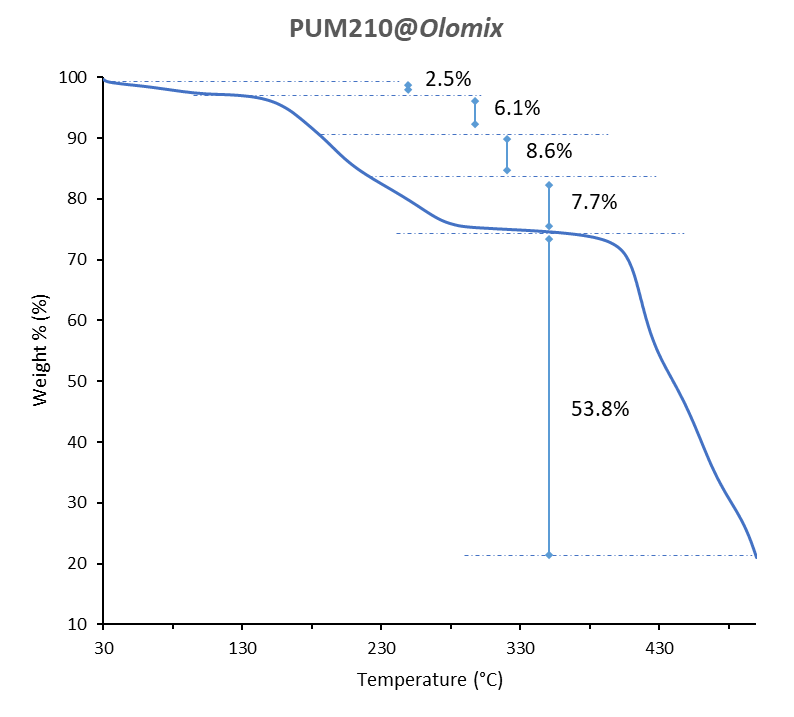


**Figure 4S.** TGA of **PUM210@*olomix*** after three days of soaking in the equimolar mixture***,*** air, 10°C/min, 30-500 °C.

## **^1^H NMR spectra of the guest uptake experiments.**


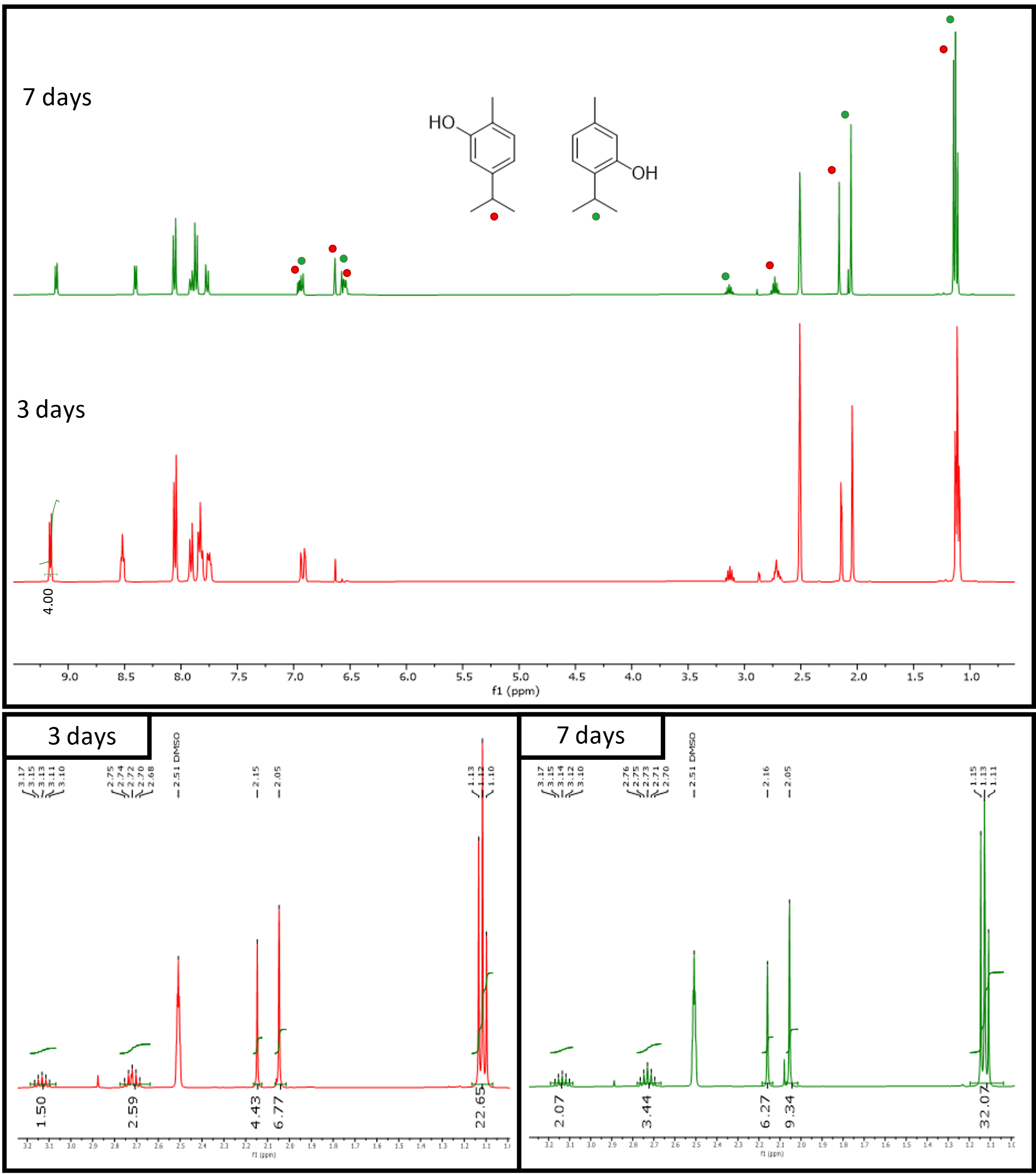


**Figure 5S.** Top: ^1^H NMR spectra of **PUM168@*olomix*** obtained at 25°C after 3 days and 7 days; TFA-d/DMSO-d_6_, 400 MHz, 25°C. The normalized peak of the pyridine is highlighted, alongside the integration value. Bottom: aliphatic window of the spectra containing the signals used for the relative quantification of the two guests. These signals were used because not affected by exchange phenomena.

1 day

2 days

5 days

7 days

1 day

2 days

5 days

7 days

1 day

2 days

5 days

7 days

1 day

2 days

5 days

7 days


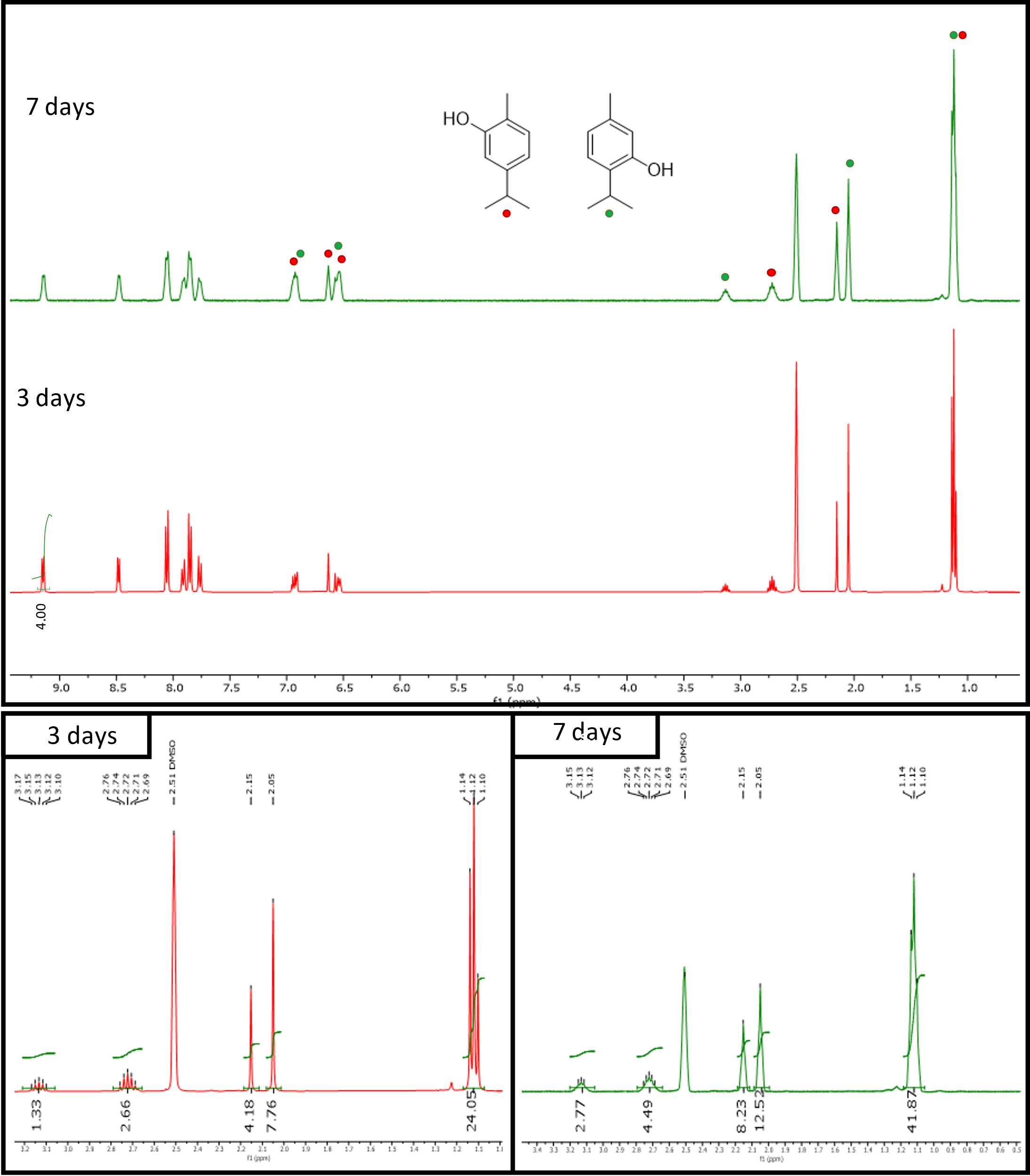


**Figure 6S.** Top: ^1^H NMR spectra of **PUM168@*olomix*** obtained at 40°C after 3 days and 7 days; TFA-d/DMSO-d_6_, 400 MHz, 25°C. The normalized peak of the pyridine is highlighted, alongside the integration value. Bottom: aliphatic window of the spectra containing the signals used for the relative quantification of the two guests. These signals were used because not affected by exchange phenomena.


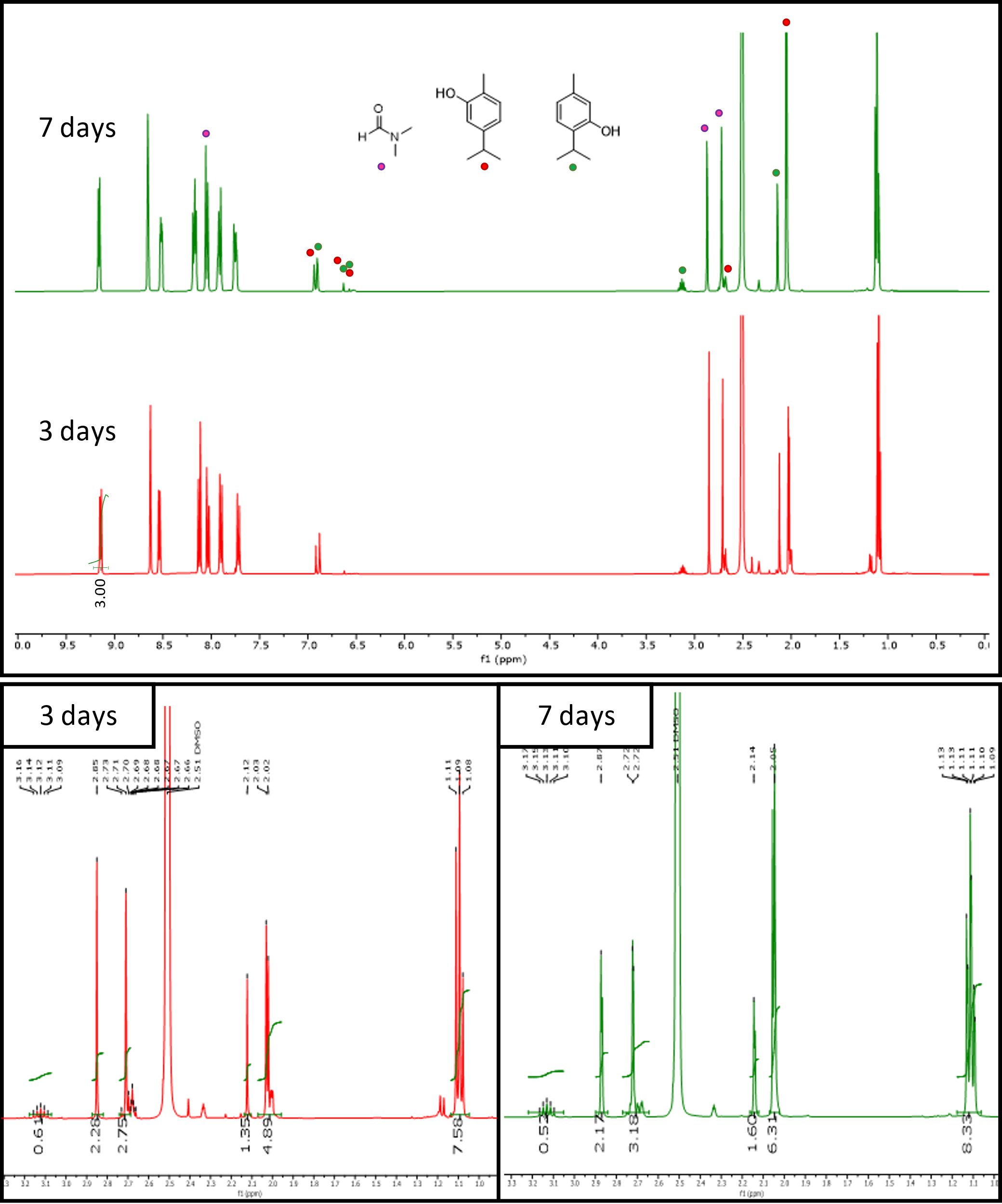


**Figure 7S.** ^1^H NMR spectra of **PUM210@*olomix*** after 3 days and 7 days of soaking at 25°C; in TFA-d/DMSO-d_6_, 400 MHz, 25°C. The normalized peak of the pyridine is highlighted, alongside the integration value. Bottom: aliphatic window of the spectra containing the signals used for the relative quantification of the two guests. These signals were used because they were not affected by exchange phenomena.


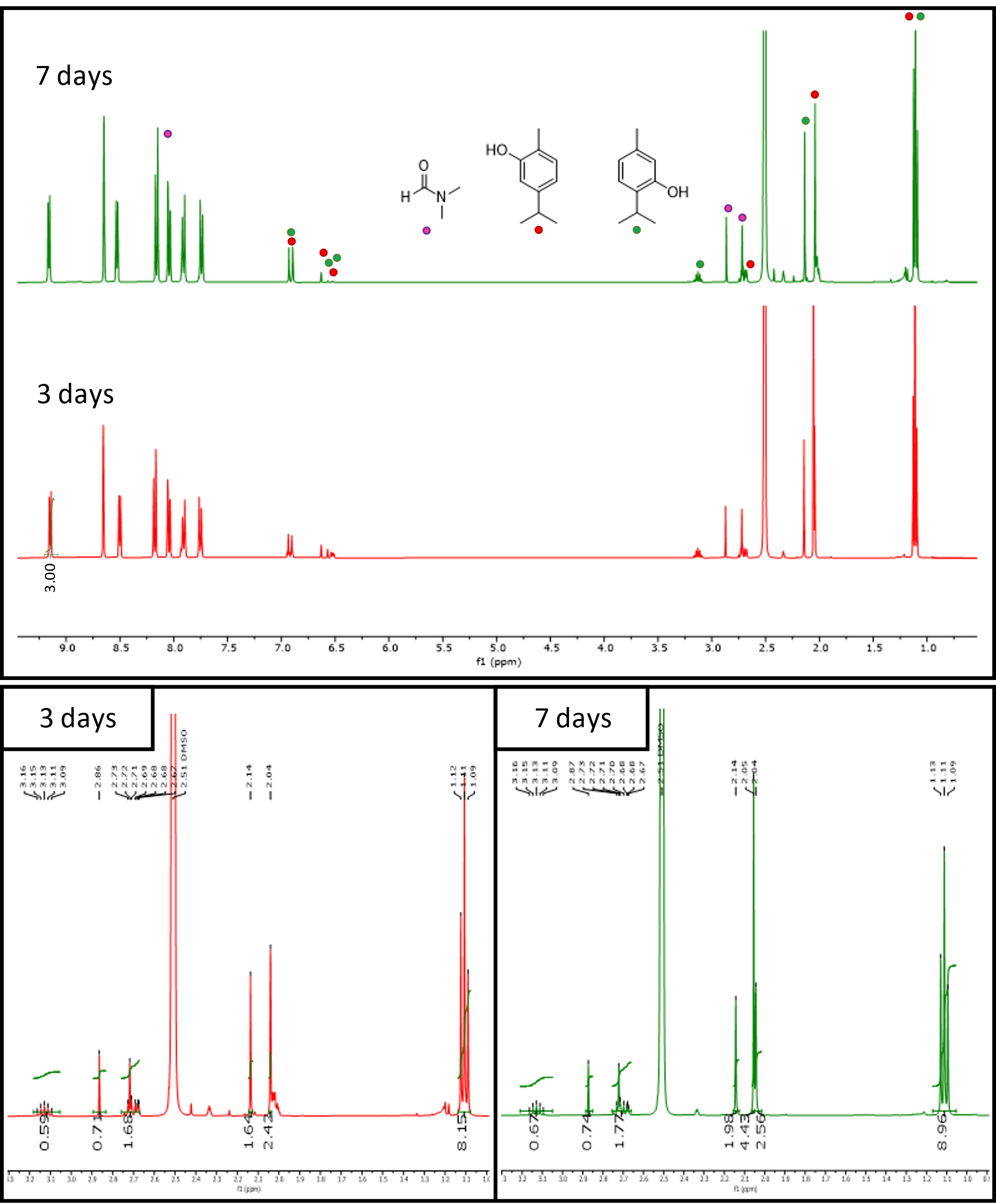


**Figure 8S.** Top: ^1^H NMR spectra of **PUM210@*olomix*** after 3 days and 7 days of soaking at 40°C; in TFA-d/DMSO-d_6_, 400 MHz, 25°C. The normalized peak of the pyridine is highlighted, alongside the integration value. Bottom: aliphatic window of the spectra containing the signals used for the relative quantification of the two guests. These signals were used because they were not affected by exchange phenomena.

# **ESEM ANALYSIS**

## **ESEM images of PUM168 after *olomix* soaking experiment**


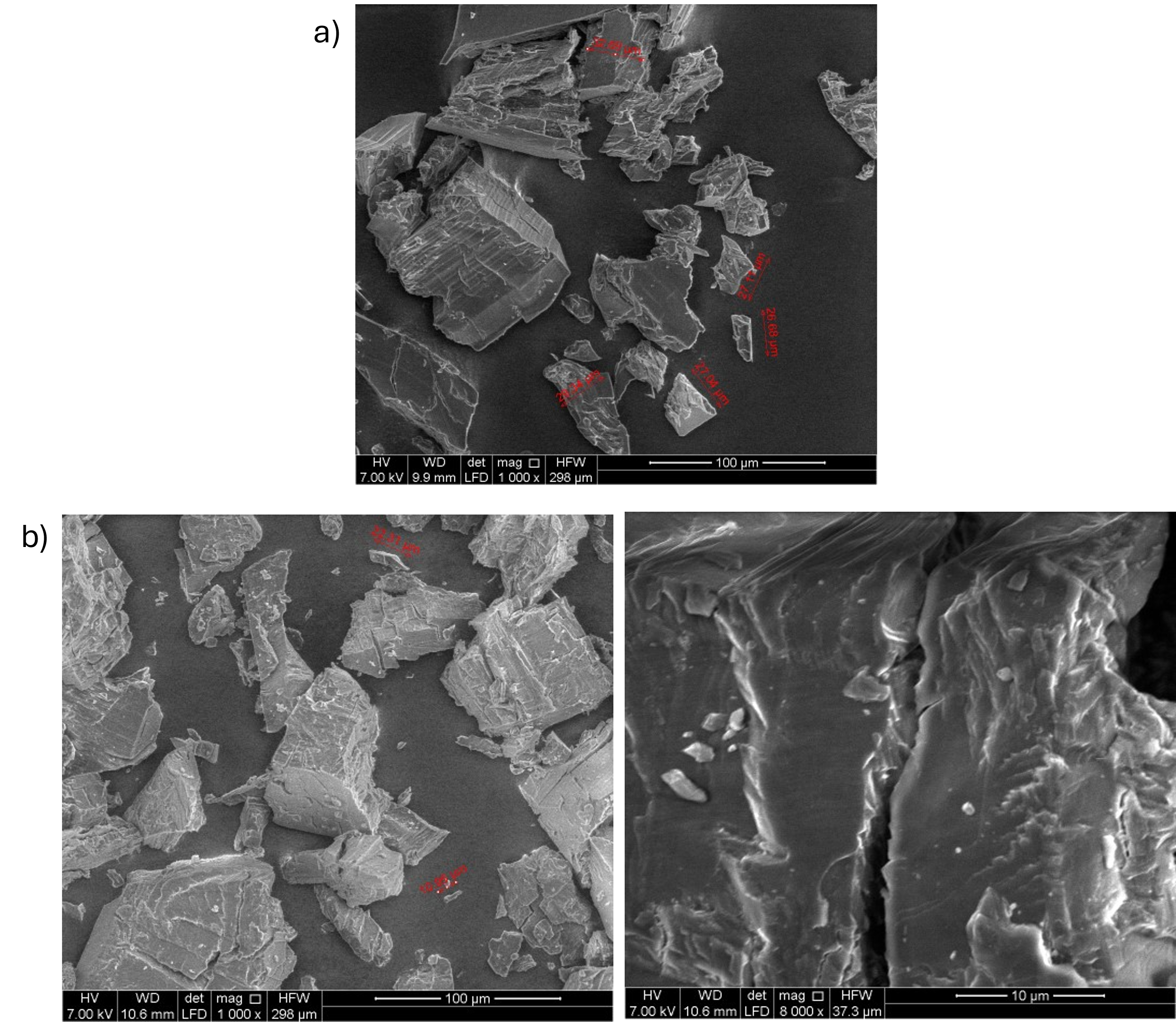


**Figure 9S. a)** Visualization of the fragmentation of PUM168 after soaking in acetone, **b)** comparison of SEM images of **PUM168@*olomix*** crystals.


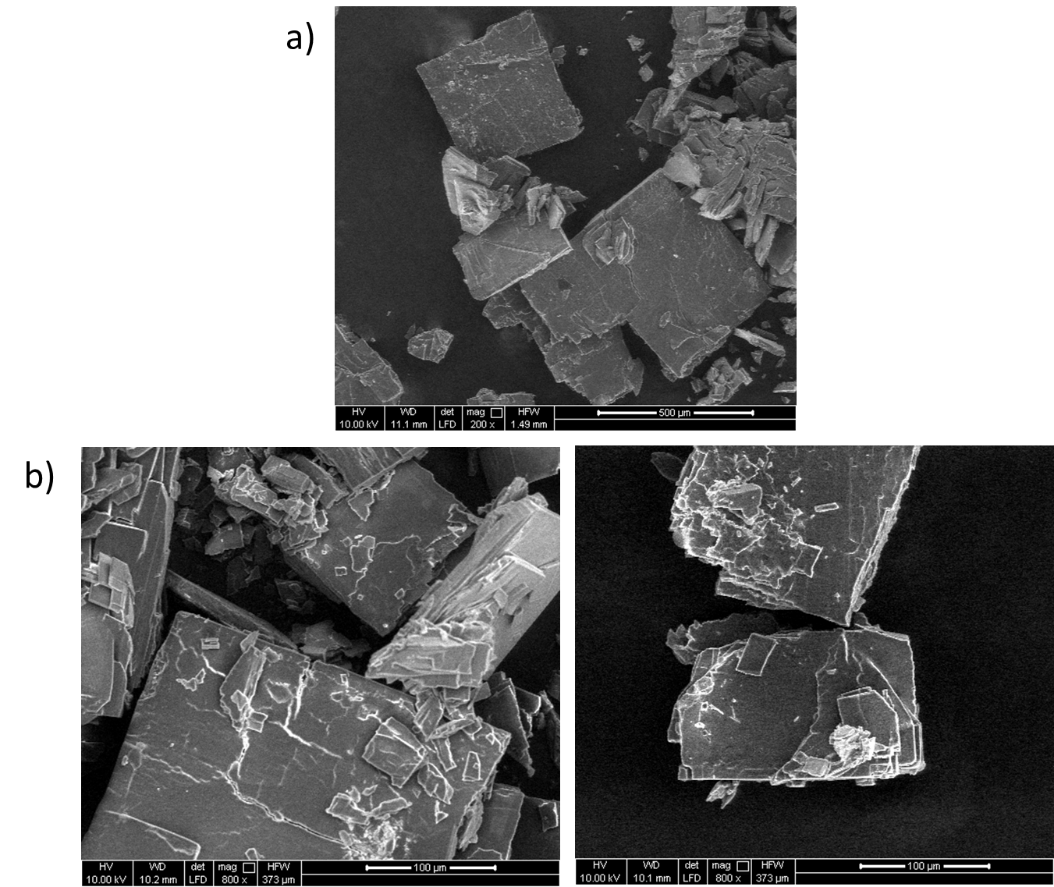


**Figure 10S. a)** Visualization of the crystals of PUM210@ACE after soaking in acetone, **b)** comparison of SEM images of **PUM210@*olomix*** crystals

# **SC-XRD ANALYSIS**

## **SC-XRD analyses on PUM210@*olomix***


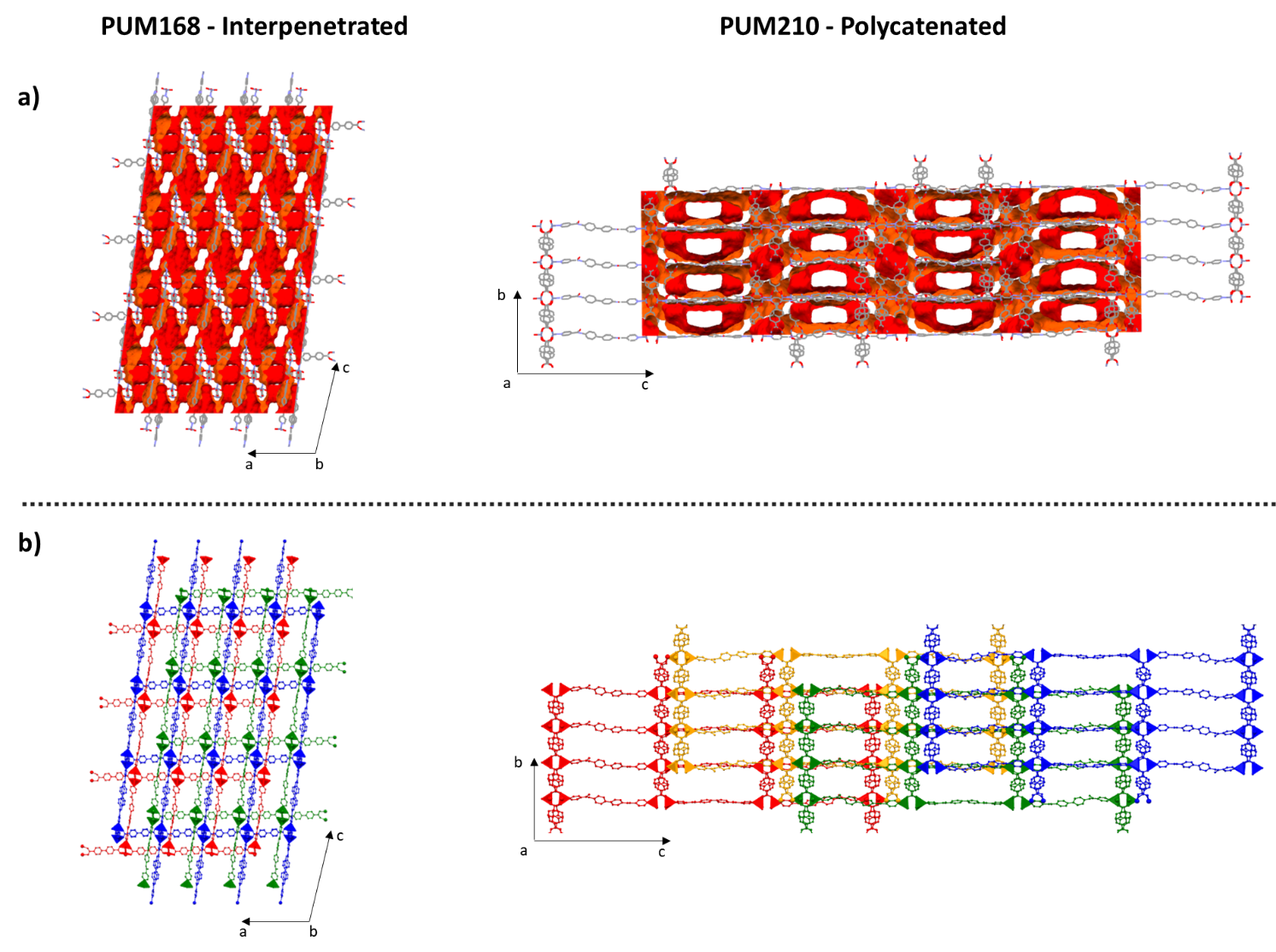


**Figure 11S.** Comparison between the structures of **PUM168@DMF** and **PUM210@DMF** to highlight the structural differences. **a)** Visualization of the calculated voids of either **PUM168@DMF** (left, view along the crystallographic axis ***b***) and **PUM210** (right, visualization along the crystallographic axis ***a***). **b)** Comparison between the two different entanglements of **PUM168@DMF** (left, visualization along the crystallographic axis ***b***) and **PUM210@DMF** (right, visualization along the crystallographic axis ***a***).


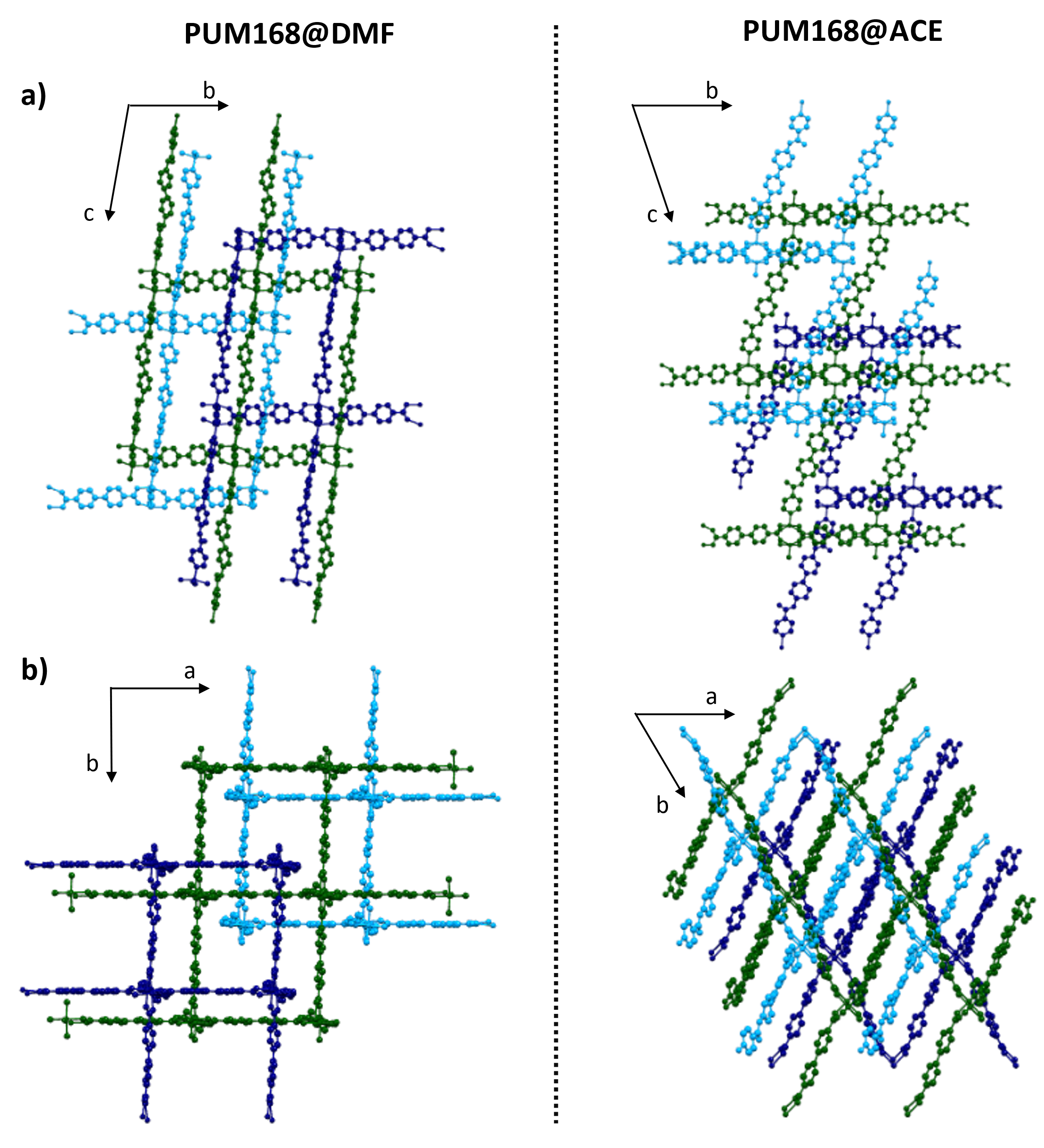


**Figure 12S.** Comparisons between the structures of **PUM168@DMF** and **PUM168@ACE** to highlight the structural deformation along **a)** crystallographic axis ***a*** and **b)** crystallographic axis ***c***.


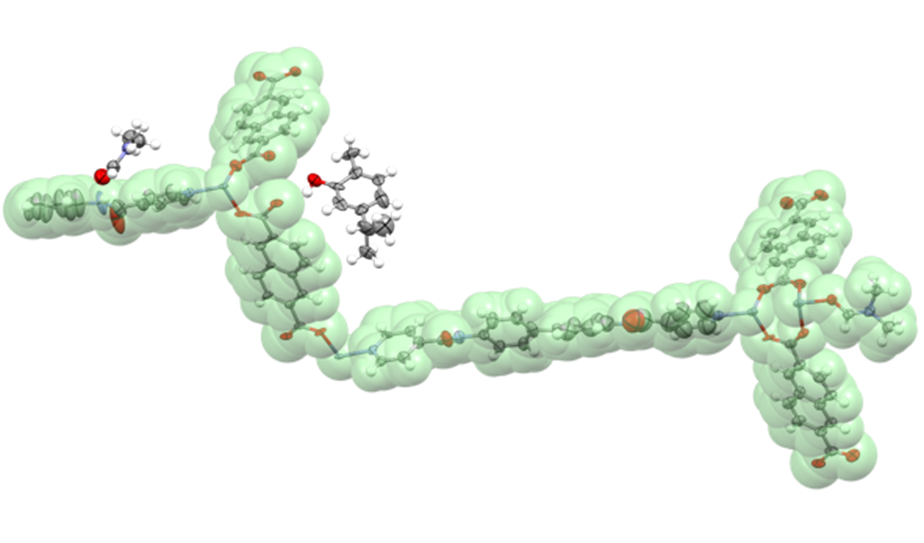


**Figure 13S.** Graphical representation of the asymmetric unit of **PUM210@*olomix***.

~~
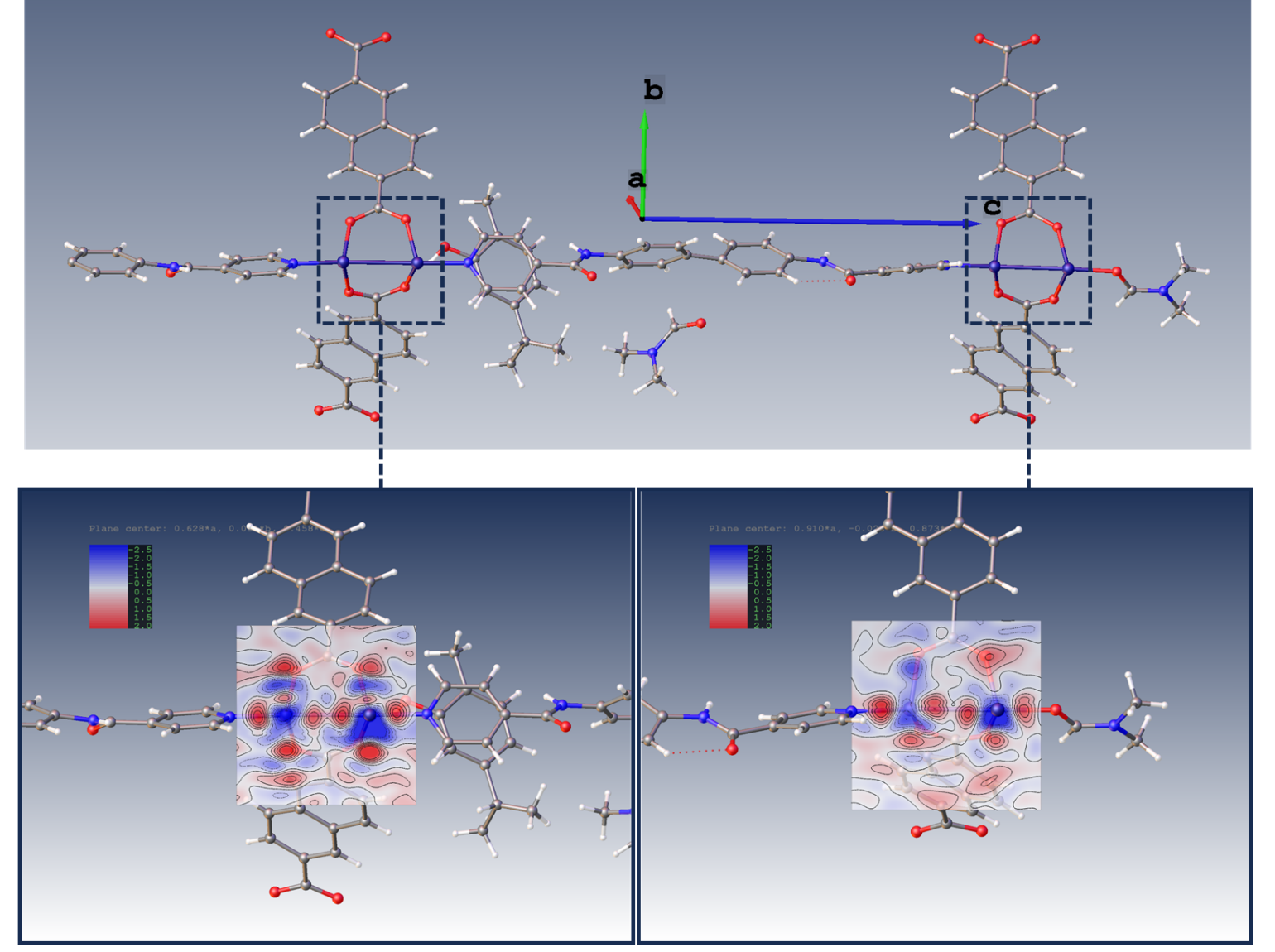
~~

**Figure 14S.** Representation of the disorder found in the structure of PUM210@*olomix*. Above: asymmetric unit of PUM210@*olomix* displayed using the GUI function of Olex2 software. Below, the calculation of the difference electron density map over the two different paddlewheel SBUs. The final R-factor is quite high with respect to the good Rmerge, and the final difference map displays very high residuals contouring the paddle-wheel SBU (about 2.5-3.5 e/A°3), while, the thermal displacement parameters of the Zn atoms and the SBU core do not show apparent anomalies. A similar situation was previously observed and discussed for native PUNM210 (see ref. 16 in the main text). There, uncorrelated displacements of the entire framework along the c axis were suggested based on the high residuals along the pillar direction, supported also by evidence of diffuse scattering along the l index. In the present case, an additional pair of high residuals appears along the ab diagonal, representing the highest residuals in the final map. The analysis of reflection statistics shows the presence of some violations of the systematic absences h0l with odd l, which weakens the space-group assignment, ascribable to the displacive disorder already described.  Moreover, the intensities analysis performed through the 'Twinning' tool in Olex2 points to a 20% fraction of twinning by 2-fold rotation along a direction slightly tilted with respect to the c axis. We conclude that the high residuals in the final map and the related high R-factor derive from the previously observed uncorrelated flexibility along the c axis convoluted with an un-modeled twinning, which altogether do not compromise the structural interpretation even if they worsen the final R-factors.


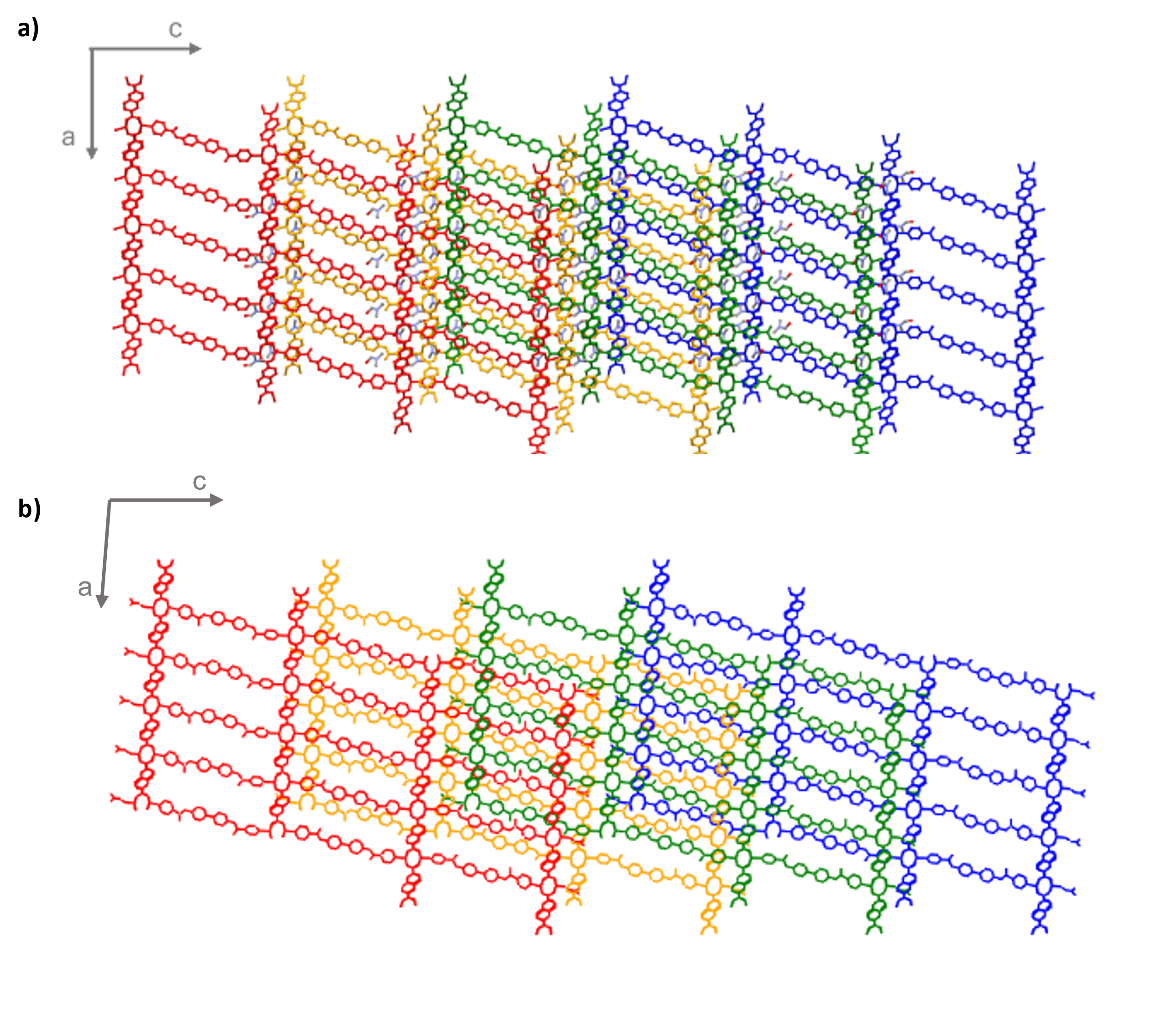


**Figure 15S.** Comparison of the frameworks of native PUM210 (top) and **PUM210@*olomix*** (bottom). Hydrogen atoms are omitted for the sake of clarity.


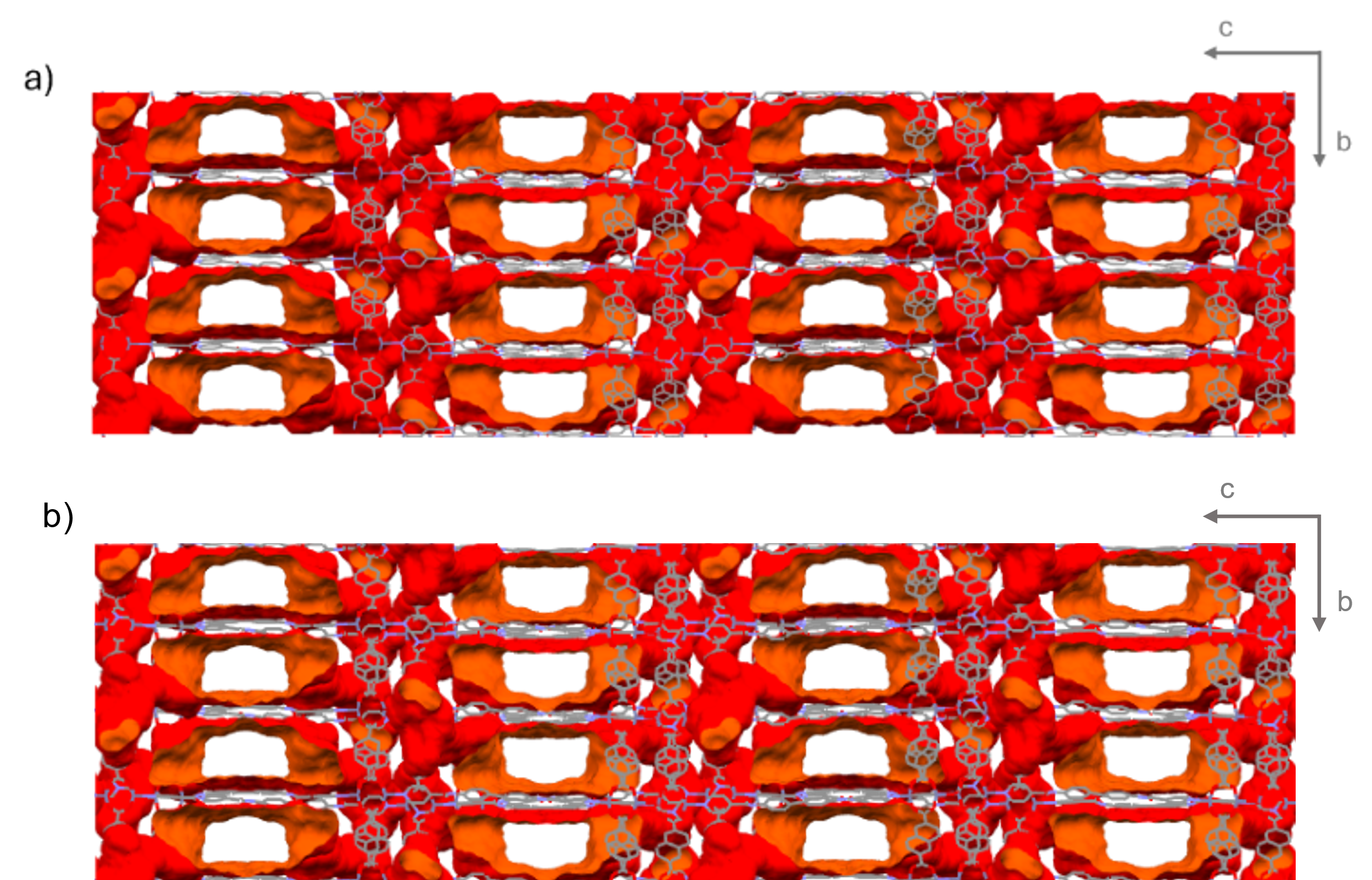


**Figure 16S.** Comparison of the calculated potential voids, after removal of guest molecules, of a) native PUM210 (41.2%, 8763.14 Å^3^) and b) ***PUM210@olomix*** (38.7%, 8535.83 Å^3^), viewed along crystallographic axis **a**. The calculation is performed using a probe radius of 1.2 Å and a grid spacing of 0.3 Å.


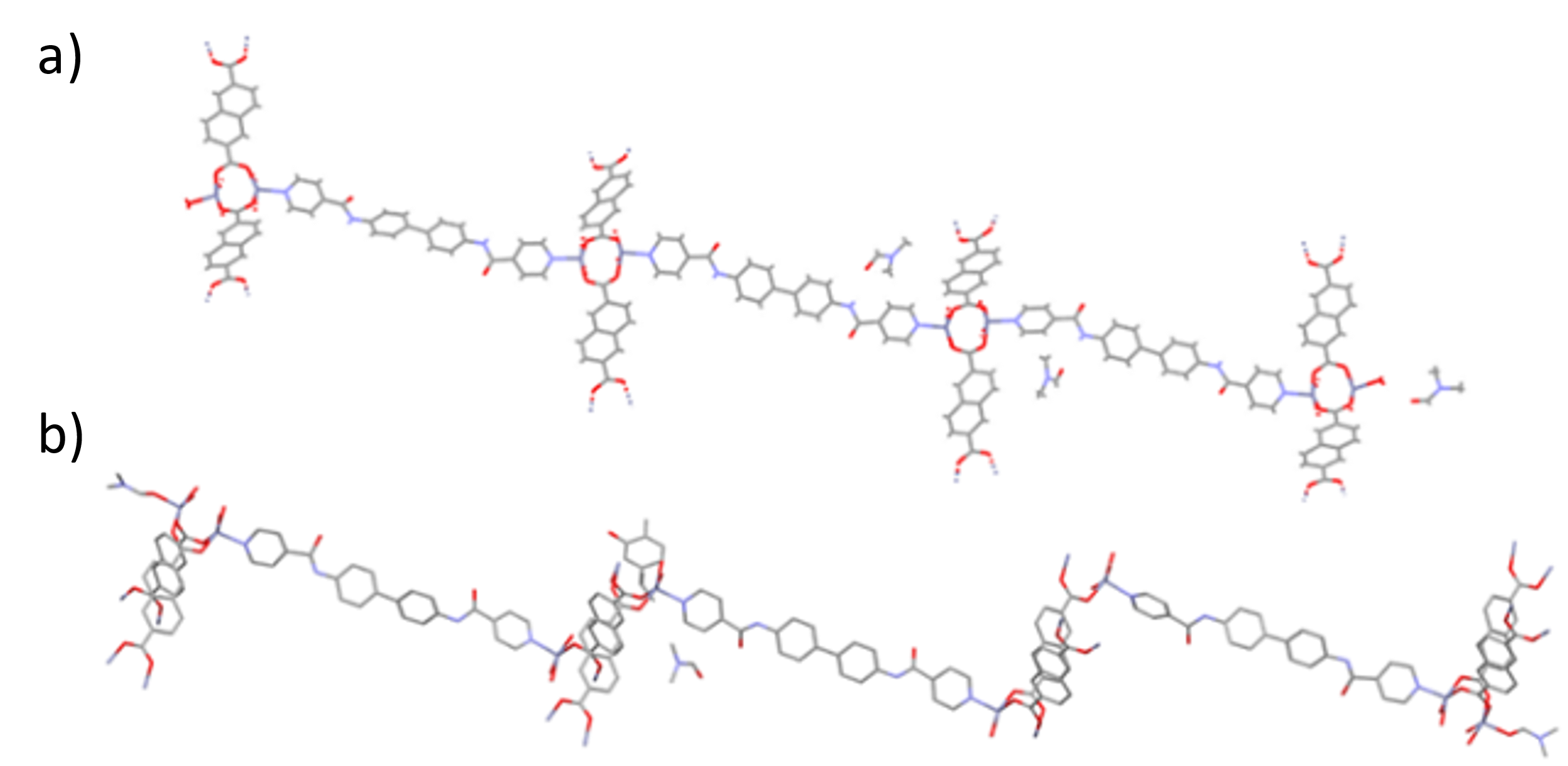


**Figure 17S.** **a)** and **b)** comparison of the relative conformations of the amide groups of L1 within the framework of pristine **PUM210** and **PUM210@*olomix*,** respectively.

# **PXRD ANALYSIS**





**Figure 18S.** Comparison between the powder X-Ray diffractogram of **PUM168@*olomix*** (displayed in red, top) and PUM168@DMF (displayed in orange, bottom).





**Figure 19S.** Comparison between the powder X-Ray diffractogram of **PUM210@*olomix*** (displayed in red, top) and PUM210@DMF (displayed in orange, bottom).





**Figure 20S.** Pawley fit of **PUM210@*olomix*** in 2 theta range 2-50. The experimental pattern is reported as a black line, fitting in red. The difference between the two patterns and the reflection position are reported below the patterns as a grey line and blue ticks, respectively.

# **HS-GC-MS RELEASE**

## **HS-GC-MS analysis: sample preparation and analytical protocol**

The **PUM168@*olomix*** and **PUM210@*olomix*** samples for HS-GC-MS analyses were prepared, in duplicate, as following:

- The excess of the olomix solution was removed and the crystals were dried under vacuum for half an hour
- MOF crystals were weight in a vial (0.5 mg for PUM168 and 0.64 mg for PUM210) so that the amount of guest contained was equal to the weighed amount of the free *olomix* components which were already prepared in relative THY:CAR ratio obtained from ^1^H NMR analysis. This ratio refers to the weight % of the two guests, as obtained from TGA
- The vials, without the cap, were left evaporating at room temperature under the hood
- The samples were capped and submitted to HS-GC-MS analysis, as a function of time after 0, 3, 7, 10 and 15 days. One milliliter of the headspace above the sample was injected into the gas chromatograph by using a PAL COMBI-xt autosampler (CTC Analytics AG, Zwingen, Switzerland). Chromatographic separation was carried out using a HP 6890 Series Plus gas chromatograph (Agilent Technologies, Palo Alto, CA) equipped with a MSD 5973 mass spectrometer (Agilent Technologies). Helium was used as the carrier gas at a constant flow of 1.3 mL/min. The following temperature program was applied to a MDN-5S 30 m × 0.25 mm, df 0.25 μm capillary column (Supelco, Bellafonte, USA): 80 °C for 0.5 min, 10 °C/min to 120 °C, 15 °C/min to 250 °C, 250 °C for 1 min. The transfer line and source were maintained at the temperatures of 270 and 150 °C, respectively. Full scan electron ionization data were acquired under the following conditions: ionization energy: 70 eV; mass range: 40−200 amu; scan time: 3 scan/s; electron multiplier voltage: 1250 V. The HP Chemstation (Agilent Technologies) was used for signal acquisition and data handling.
- In parallel, 0.16 µL of *olomix* were weighted in duplicate and processed under the same conditions (THY: CAR ratio of 1:1.9, respectively. The relative ratio was estimated through ^1^H NMR analysis and corresponds to the equivalent amount present within the MOF)

**Table 1S.** HS-GC-MS release (mean response ± std.dev, n=2) of THY and CAR from: **A)** **PUM168@*olomix*** and **B)** pure *olomix* as a function of time (days).

**A)**

| **THYMOL CARVACROL** | | |
| --- | --- | --- |
| **Time (day)** | **Area ± Std. dev.** | **Area ± Std. dev.** |
| **0** | 1100±300 | 320±70 |
| **3** | 138.9±4.4 | 47.5±0.1 |
| **7** | 143±6 | 50±6 |
| **10** | 130±10 | 56.4±0.2 |
| **15** | 160±30 | 70±10 |

**B)**

| **THYMOL CARVACROL** | | |
| --- | --- | --- |
| **Time (day)** | **Area ± Std. dev.** | **Area ± Std. dev.** |
| **0** | 15000±100 | 5900±100 |
| **3** | - | - |
| **7** | - | - |
| **10** | - | - |
| **15** | - | - |

- Not detected

**Table 2S.** HS-GC-MS release (mean response ± std.dev, n=2) of THY and CAR from: **A)** **PUM210@*olomix*** and **B)** pure *olomix* as a function of time (days).

**A)**

| **THYMOL CARVACROL** | | |
| --- | --- | --- |
| **Time (day)** | **Area ± Std. dev.** | **Area ± Std. dev.** |
| **0** | 80±20 | 200±30 |
| **3** | - | - |
| **7** | - | - |
| **10** | - | - |
| **15** | - | - |

- Not detected

**B)**

| **THYMOL CARVACROL** | | |
| --- | --- | --- |
| **Time (day)** | **Area ± Std. dev.** | **Area ± Std. dev.** |
| **0** | 11500±300 | 5700±100 |
| **3** | - | - |
| **7** | - | - |
| **10** | - | - |
| **15** | - | - |

- Not detected

**Crystallographic table**

**Table 3S.** Crystallographic data for **PUM210@*Olomix***

| **PUM210@olomix** | |
| --- | --- |
| Empirical formula | C_217_H_179_N_15_O_45_Zn_8_ |
| Formula weight | 4239.70 |
| Temperature/K | 200.15 |
| Crystal system | monoclinic |
| Space group | C2/c |
| a/Å | 19.0583(5) |
| b/Å | 18.1097(5) |
| c/Å | 64.1607(14) |
| α/° | 90 |
| β/° | 95.559(2) |
| γ/° | 90 |
| Volume/Å^3^ | 22040.3(10) |
| Z | 4 |
| ρ_calc_g/cm^3^ | 1.278 |
| μ/mm^‑1^ | 1.565 |
| F(000) | 8744.0 |
| Crystal size/mm^3^ | 0.022 × 0.02 × 0.018 |
| Radiation | CuKα (λ = 1.54184) |
| 2Θ range for data collection/° | 5.536 to 140.118 |
| Index ranges | -22 ≤ h ≤ 22, -22 ≤ k ≤ 22, -77 ≤ l ≤ 77 |
| Reflections collected | 79610 |
| Independent reflections | 20062 [R_int_ = 0.0762, R_sigma_ = 0.0593] |
| Data/restraints/parameters | 20062/2088/1124 |
| Goodness-of-fit on F^2^ | 2.577 |
| Final R indexes [I>=2σ (I)] | R_1_ = 0.2128, wR_2_ = 0.5593 |
| Final R indexes [all data] | R_1_ = 0.2169, wR_2_ = 0.5635 |
| Largest diff. peak/hole / e Å^-3^ | 3.63/-3.91 |
|  |  |
